# Synthesis and immunological evaluation of protein conjugates of *Neisseria meningitidis* X capsular polysaccharide fragments

**DOI:** 10.3762/bjoc.10.247

**Published:** 2014-10-13

**Authors:** Laura Morelli, Damiano Cancogni, Marta Tontini, Alberto Nilo, Sara Filippini, Paolo Costantino, Maria Rosaria Romano, Francesco Berti, Roberto Adamo, Luigi Lay

**Affiliations:** 1 Dipartimento di Chimica and ISTM-CNR, Universita degli Studi di Milano, via Golgi 19, I-20133 Milano, Italy; 2 Novartis Vaccines, Via Fiorentina 1, 53100 Siena, Italy

**Keywords:** carbohydrates, glycoconjugates, immunology, multivalent glycosystems *Neisseria meningitidis*, vaccines

## Abstract

A vaccine to prevent infections from the emerging *Neisseria meningitidis* X (MenX) is becoming an urgent issue. Recently MenX capsular polysaccharide (CPS) fragments conjugated to CRM_197_ as carrier protein have been confirmed at preclinical stage as promising candidates for vaccine development. However, more insights about the minimal epitope required for the immunological activity of MenX CPS are needed. We report herein the chemical conjugation of fully synthetic MenX CPS oligomers (monomer, dimer, and trimer) to CRM_197_. Moreover, improvements in some crucial steps leading to the synthesis of MenX CPS fragments are described. Following immunization with the obtained neoglycoconjugates, the conjugated trimer was demonstrated as the minimal fragment possessing immunogenic activity, even though significantly lower than a pentadecamer obtained from the native polymer and conjugated to the same protein. This finding suggests that oligomers longer than three repeating units are possibly needed to mimic the activity of the native polysaccharide.

## Introduction

*Neisseria meningitidis* is an encapsulated, aerobic gram-negative diplococcus which causes significant morbidity and mortality in newborns, children and young adults worldwide through meningitis and/or septicemia. Although sporadic cases occur in Europe and North America, major meningitis epidemics have been recorded in Africa, in an area termed “the meningitis belt”, extending from Senegal to Ethiopia and including 21 countries with a population of over 300 million people. According to the chemical composition of the bacterial capsular polysaccharide (CPS) [1], 13 serogroups of *N. meningitidis* have been so far defined. Until recently only five of them (A, B, C, Y, W135) were associated with significant pathogenic potential [2–3]. In particular serogroup A (MenA), that caused significant meningitis outbreaks in industrialised countries until the 1970s [4], is currently a major responsible for epidemics in the African meningitis belt. Additionally, since 2002 serogroup W135 has also been considered a major threat. In the past 20 years sporadic cases or clusters of meningitis due to other *N. meningitidis* serogroups have emerged. While the impact of infections caused by serogroup X of *N. meningitidis* (MenX) was initially considered negligible, in the last decade incidence rates and disease characteristics very similar to other virulent meningococcal isolates have been reported. As a matter of fact, the meningitis cases ascribed to MenX do not present any clinical or epidemiological differences to those due to serogroup A. Most cases (93%) were recorded during the dry season, with a mean age of the patients of 9.2 years and a fatality rate of 11.9% [5]. MenX was first described in the 1960s [6], when it was found to cause a few cases of invasive disease across North America, Europe, Asia and Africa [7]. The first case of MenX disease in Africa was documented in 1974 and, since then, several sporadic cases have been observed in other African countries [8]. In 2006, the occurrence in Niger of MenX related meningitis infections with unprecedented incidence led the World Health Organization (WHO) to consider MenX as a substantial threat [9]. However, it was only in 2010 that, following a very large MenX outbreak in Burkina Faso, the WHO-Inter-country Support Team (WHO-IST) weekly bulletins on meningitis started to specifically document MenX epidemics [10]. Interestingly, while MenA incidence decreased in most meningitis belt countries following the introduction in 2010 of a monovalent MenA conjugate vaccine (MenAfriVac) [11–12], an increase in MenX cases has been observed. Recently a study revealed that in Burkina Faso the levels of MenX carriage after the introduction of the MenA conjugate vaccine are significantly higher than they were before the vaccine introduction [13]. This could suggest a serotype replacement due to mass vaccination with MenAfriVac, although this event should be considered unlikely on the basis of previous experiences with the introduction of the MenC conjugate vaccine [14]. Undoubtedly the recent increase of MenX infections has led to take in consideration this emerging serogroup for the development of new meningococcal vaccines [15–16]. Recently it has been reported that coupling long chain oligosaccharides from MenX CPS to the nontoxic mutant of diphtheria toxin Cross-Reacting Material 197 (CRM_197_), a protein widely used in manufactured vaccines,[17] provides a potent candidate for the development of a glycoconjugate vaccine against this serogroup [18].

**Figure 1:**
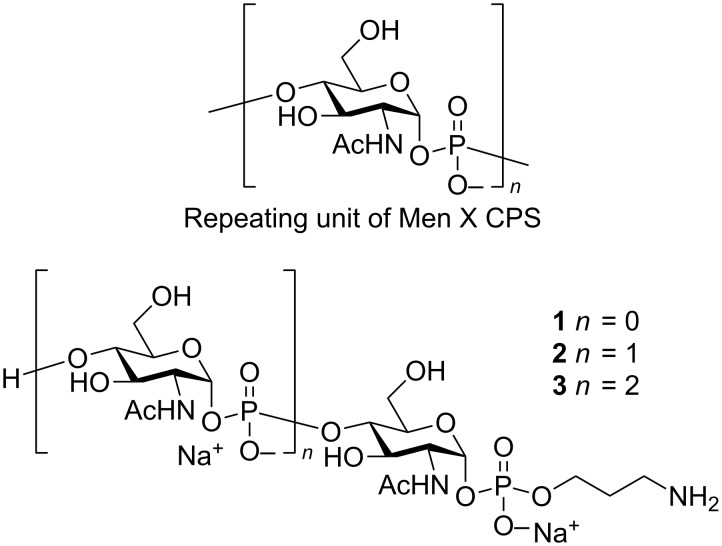
Structures of the repeating unit of MenX CPS and synthetic oligomers **1–3**.

MenX CPS is a homopolymer of (1→4)-linked 2-acetamido-2-deoxy-α-D-glucopyranosyl phosphate residues (Figure 1). The synthesis of the repeating unit was first reported in 1974 [19], and more recently an improved protocol for larger scale preparation of the monomer as analytical tool has been also described [20]. Notably, the minimal CPS portion which can confer protection against meningococcal infections is still unknown.

Over the recent years, advances in the synthesis of complex glycans are rendering accessible a variety of carbohydrate antigens with well-defined chemical structure and devoid of bacterial contaminations which could derive from purification of biological materials [21–24]. This could be a crucial feature to improve batch-to-batch consistency in vaccine manufacturing and to confer a better safety profile.

Some of us have recently achieved the first synthesis of short-chain MenX CPS oligomers (compounds **1**–**3**, Figure 1) provided with a phosphodiester-linked aminopropyl spacer suitable for their conjugation to a carrier protein [25].

These synthetic molecules are valuable tools to obtain information on the minimal structural requirements for the immunological activity of MenX CPS and for evaluation as vaccine candidates. In the present work we report the preparation and in vivo immunological evaluation of neo-glycoconjugates from the fragments **1**–**3.** In this context, we describe the significant improvements recently achieved in some crucial steps of our previously reported synthesis [25] that will render more expeditious the preparation of this type of oligomers.

The synthetic oligomers **1**–**3** were conjugated to the surface abundant lysine residues of the carrier protein CRM_197_ by means of the di(*N*-succinimidyl) adipate (SIDEA) linker. We have already shown that this spacer, which is used in commercial anti-meningococcal vaccines for the feature of being immuno-silent, can be efficiently utilized for the conjugation of short synthetic antigens bearing an amino spacer [26–27]. The synthesized CRM_197_ glycoconjugates were first tested for their capability of eliciting anti MenX CPS antibodies in mice. The functional activity of the generated antibodies was then assessed by the in vitro bactericidal assay recently developed for the evaluation of MenX CPS conjugates [18].

## Results and Discussion

### Improvements in α-H-phosphonate synthesis

Our previous synthesis of oligomers **1**–**3** featured the use of 2-azido-2-deoxy glucopyranosyl building blocks and their corresponding glycosyl hydrogenphosphonates (H-phosphonates) intermediates for the installation of the phosphodiester linkages [28].

We were confident that the use of the non-participating and electron-withdrawing azido group could strongly enhance the stability of the anomeric phosphodiester linkages. However, we experienced a dramatic drop of the overall yield during the conversion of the azido groups in acetamides on protected glycosyl phosphosaccharide intermediates [25].

**Scheme 1:**
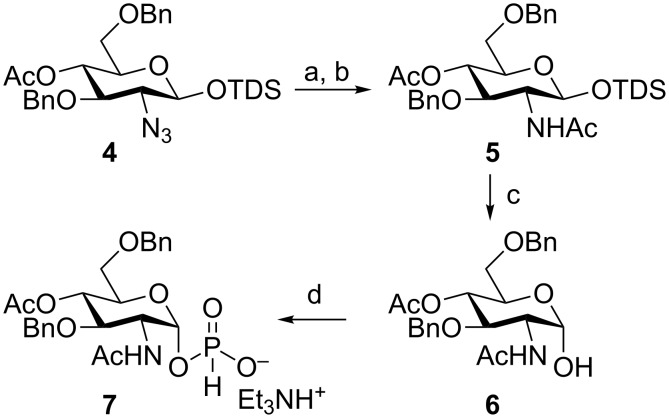
Reagents and conditions: a) NiCl_2_/NaBH_4_, MeOH; b) Ac_2_O, 86% over 2 steps; c) TBAF, THF, −40 °C to rt, 81%; d) salicylchlorophosphite, pyridine, then 1M triethylammonium hydrogencarbonate buffer solution (TEAB): Batch 62% yield; MRT 76% yield. TDS: Thexyldimethylsilyl.

We therefore sought to design a different strategy based on GlcNAc instead of azido glucose building blocks, where the azide reduction is rather performed at an early stage of the synthetic route than on valuable advanced intermediates. Accordingly, the azide reduction with NiCl_2_/NaBH_4_ protocol [29] occurred smoothly on the previously described [25] silyl glycoside **4**, and after standard N-acetylation furnished acetamide **5** in high yield (Scheme 1).

On the other hand, the same reaction carried out on protected glycosyl phosphosaccharides afforded the corresponding acetamides in 25–35% yield [25]. Interestingly, when compound **5** was subjected to 1-O-desilylation with tetrabutylammonium fluoride in THF at –40 °C, we obtained exclusively the α-hemiacetal **6** in 81% yield (Scheme 1). The formation of the α anomer was confirmed by the doublet of H-1 at 5.23 ppm in the ^1^H NMR spectrum with the typical value of ^1^*J*_1,2_ = 3.5 Hz, and the appearance of the C-1 signal at 92.0 ppm in the ^13^C NMR spectrum (see Supporting Information File 1).

Most importantly, when the hemiacetal **6** was treated with salicylchlorophosphite in pyridine at room temperature the α-H-phosphonate **7** was obtained as a single anomer in only 2 h in 62% yield. We reasoned that the occurrence of an intramolecular hydrogen bond involving the acetamido group could be the main responsible for the high selectivity observed in the formation of compound **6** and, consequently, for the attainment of the pure α-H-phosphonate **7**. Indeed, the desilylation of the 2-azido counterpart of intermediate **5** provided a mixture of anomers. On the other hand, the same reaction carried out on a 2-acetamido derivative very similar to **5** but protected as a 4,6-*O*-benzylidene acetal also led to an anomeric mixture, suggesting that conformational factors might be also involved. In addition, the treatment of this mixture with salicylchlorophosphite produced a mixture of anomeric H-phosphonates, indicating that no equilibration of the anomers occurs during this reaction. We, therefore, ascribed the high stereoselectivity observed in the formation of compound **7** to the stability of compound **6**, whose configuration is preserved during the reaction with salicylchlorophosphite. This finding introduced a great improvement in our reported synthesis of MenX CPS oligomers, since in the previous protocol extremely long reaction times (6–7 days) were needed for the exclusive formation of the most thermodynamically stable α-H-phosphonate by equilibration in the presence of H_3_PO_3_ of the initially formed mixture of anomeric H-phosphonates [25]. An additional improvement in α-H-phosphonate **7** formation was achieved by carrying out the reaction under microfluidic conditions. The Micro Reactor Technology (MRT) is gaining increasing attention for drug discovery. Some of its various possible advantages when compared to more conventional approaches are improved safety characteristics, enhanced rates of heat and mass transfer, simplicity and robustness in scale-up and easiness in handling the instrumentation [30–33]. For the synthesis of compound **7**, two distinct solutions containing the hemiacetal **6** in pyridine and salicylchlorophosphite in CH_3_CN, respectively, were pumped in a 100 μL glass microreactor. The device was completed by a reservoir connected to the outlet of the microreactor, refilled with a solution of triethylammonium bicarbonate buffer (TEAB) 1.0 M to stabilize the H-phosphonate product. Setting the residence time to 3 min, 46 mg of the α-H-phosphonate **7** were obtained in 0.5 h at higher isolated yield (76%) and purity than batch reaction. Based on the maximum volume of the syringes employed in our continuous-flow system (5 mL, see Supporting Information File 1), we can estimate that a production rate of 2.2 g/day would be achievable. To the best of our knowledge, this is the first example of the synthesis of glycosyl H-phosphonates using the continuous-flow MRT [30–33].

The occurrence of the H-phosphonate **7** was ascertained by a signal at 1.87 ppm in the ^31^P NMR, and the presence in the ^1^H NMR of the diagnostic doublet at 6.93 ppm with the characteristic value of ^1^*J*_H,P_ = 631.8 Hz, typical of this class of compounds [34]. The α-configuration of the anomeric carbon was confirmed by a doublet of doublet at 5.53 ppm, with ^1^*J*_1,2_ = 3.2 Hz, ^1^*J*_1,P_ = 8.4 Hz (see Supporting Information File 1).

**Scheme 2:**
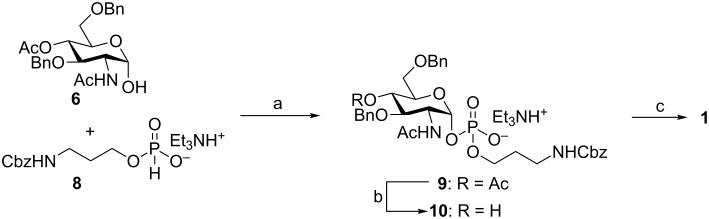
Reagents and conditions: a) PivCl in pyridine, then I_2_ in 19:1 pyridine/H_2_O, then 1 M TEAB (45%); b) NaOMe, MeOH; c) H_2_, Pd/C, MeOH/H_2_O, then H_2_O, Dowex 50W X8 resin (H^+^ form), then Dowex 50W X8 resin (Na^+^ form) (96% over two steps).

The benefit of the easy availability of the α-hemiacetal **6** was illustrated by the improved synthesis of the spacer-linked MenX monomer **1** (Scheme 2). The PivCl-mediated coupling of **6** with compound **8** [35–36] provided the glycosylphosphodiester **9** as a pure α-anomer, demonstrating the configurational stability of the hemiacetal under these reaction conditions. Compound **9** was subjected to Zemplén transesterification with NaOMe in methanol to afford alcohol **10**, and the hydrogenolytic removal of the remaining protective groups furnished the spacer-linked monomer **1** in excellent yield (Scheme 2).

### Chemical synthesis of neo-glycoconjugates

**Scheme 3:**
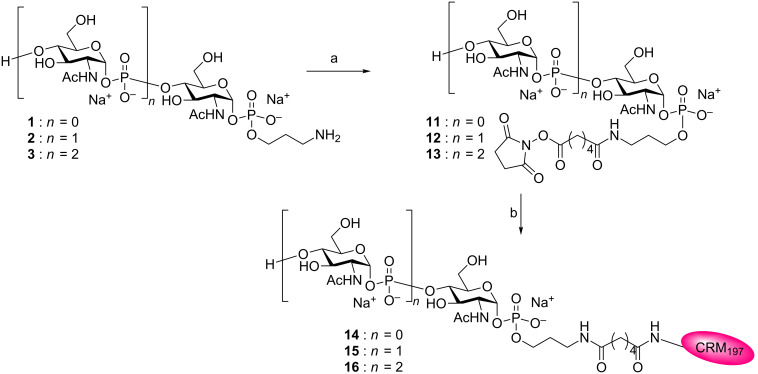
Reagents and conditions: a) SIDEA, Et_3_N, DMSO: **11** (64%), **12** (49%), **13** (51%); b) CRM_197_, 100 mM NaP_i_ pH 7.2.

Fragments **1**–**3**, obtained as previously reported [25], were employed as follows for the synthesis of the corresponding CRM_197_ conjugates. First the oligomers **1**–**3** were activated by reaction with an excess of SIDEA in the presence of triethylamine in DMSO (Scheme 3). The products were purified by precipitation from ethyl acetate, and after freeze-drying the half esters **11**–**13** were obtained at 49–65% yield. Of note, while we have utilized a similar procedure for fast and efficient insertion of the monoester of the immunosilent adipate linker onto a number of different length glycans [26–27], lower yields were attained in the present case. This can be explained with the higher solubility in organic solvents of the short structures **11**–**13** employed in the present study in comparison to other reported oligosaccharides [26–27], which did not allow complete precipitation of the activated oligomers. To increase the yield of this step, compounds **11**–**13** were recovered from the dimethylsulfoxide–ethyl acetate mixture by evaporation of ethyl acetate and addition of fresh ethyl acetate at 0 °C. The newly precipitated activated carbohydrates were freeze-dried and coupled to the protein. In this way, almost quantitative recovery of the activated sugars **11**–**13** was achieved.

Active esters **11**–**13** were then coupled with the amino groups of the protein in sodium phosphate buffer (100 mM NaPi, pH 7.2) at room temperature for 24 h (Scheme 3).

The glycoconjugates **14**–**16** were purified from the excess of unconjugated carbohydrate by precipitation with ammonium sulfate and reconstitution in 10 mM NaP_i_ pH 7.2. The occurrence of conjugation was assessed by SDS-PAGE (sodium dodecyl sulfate polyacrylamide gel electrophoresis) and MALDI–TOF mass spectrometry (see Supporting Information File 1). The latter analytical technique enabled determination of the saccharide/protein molar ratio (saccharide loading). The characteristics of the prepared glycoconjugates are summarized in Table 1.

**Table 1:** Characteristics of the synthesized glycoconjugates.

Glyco conjugate	Activated ester/protein (mol/mol)^a^	Saccharide conjugation (mol/mol)^b^	MW (Da)	Loading efficiency %^a^

**14**	75:1	7	61523	9
**15**	75:1	4.5	61731	6
**16**	75:1	4.7	63256	6
MenXDP15CRM_197_ **17**	13:1	2.5	n.d.	19

^a^Mol of activated glycan: mol of protein used in the conjugation reaction. ^b^Sugar:protein molar ratio determined by MALDI–TOF MS for **14**–**16**, and by HPAEC-PAD analysis for MenXDP15-CRM_197_ conjugate **17**.

A moderate loading (5–7 sugars/protein) was obtained in compounds **14**–**16** in respect to conjugates prepared by the same conjugation chemistry and different carbohydrate structures [26–27]. However, it needs to be taken in consideration that this loading is comparable to that achieved in the preparation of anti-meningococcal vaccines commercially available [37]. Furthermore, a number of 2.5 and 1.7–4.1 sugar moieties were incorporated in our positive control **17** and in the MenX CPS glycoconjugates recently reported to induce protective antibodies, respectively [18]. Thus, we deemed the loading of glycoconjugates **14**–**16** sufficient to determine in vivo their capability of eliciting anti-MenX CPS antibodies.

### Immunological evaluation of CRM_197_ conjugates

To evaluate the immunogenicity of the synthesized glycoconjugates, groups of 8 BALB/c mice were immunized with three doses (two weeks apart) of 0.3 μg on saccharide base of the neo-glycoconjugates. The conjugated trimer **16** was also injected at 1 μg carbohydrate base dose to evaluate the effect of dose variation. The conjugates were formulated with aluminum phosphate, an adjuvant commonly used for vaccines in the market or in preclinical development [38]. As a control, the CRM_197_ conjugate with MenX fragments having an average degree of polymerization (avDP) of 15 (MenXDP15-CRM conjugate, compound **17**) was used at the saccharide base doses of 0.3 and 1 μg, respectively.

**Figure 2:**
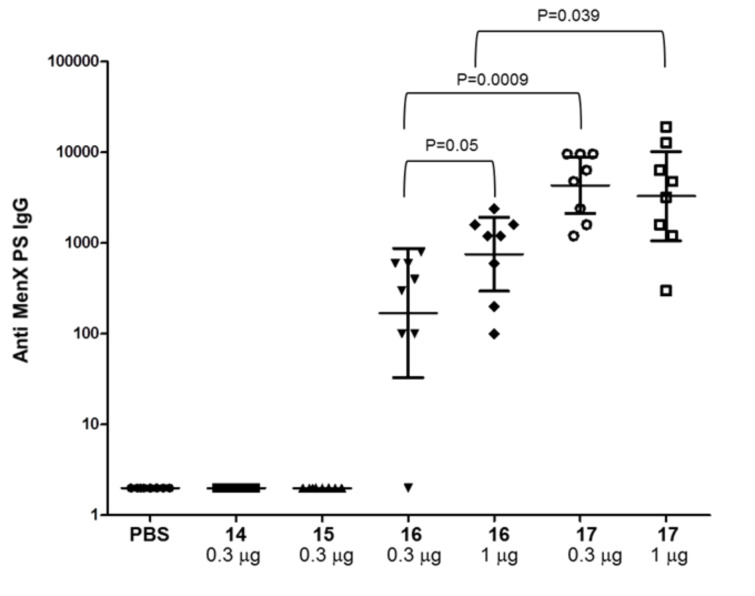
IgG levels detected at OD = 1 in individual post 3 sera (sera collected two weeks after the third immunization) of BALB/c mice immunization at 0.3 or 1 μg saccharide dose of antigen against MenX CPS as coating plate. Each dot represents individual mouse sera; horizontal bars indicate geometric mean titers (GMT) of each group with 95% statistical confidence intervals indicated by upper and lower bars.

As shown in Figure 2, while the CRM_197_ conjugates of the monomer **1** and the dimer **2** did not induce polysaccharide specific IgG titers, the conjugated trimer **3** elicited anti-MenX CPS IgG titers, with no statistical difference (p 0.05) at the doses of 0.3 and 1 μg, respectively.

The MenXDP15 conjugate **17** also induced anti-MenX CPS antibodies that were comparable each other at the two different doses (p 0.05). However, the IgG levels induced by the trimer conjugate **16** at both 0.3 and 1 μg dose were significantly lower than those elicited by the conjugate **17** administered at the corresponding doses (p 0.0009 and 0.039 for 0.3 and 1 μg dose, respectively). Importantly, all the conjugates induced very low anti-MenX IgM titers, but the trimer **3** and the MenXDP15 antigens conjugated to the carrier enabled switching from IgM to IgG, which is characteristic of the T cell dependent response (for IgM levels see Supporting Information File 1).

**Figure 3:**
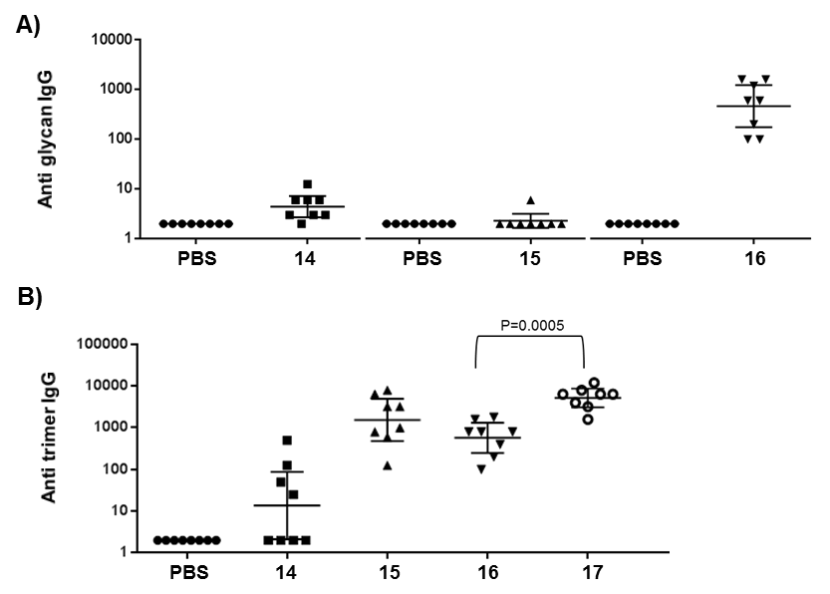
A) IgG levels detected at OD = 1 in individual post 3 sera of BALB/c mice immunization at 0.3 μg saccharide dose of antigen. A) Sera from conjugates **14***,* **15** and **16** were tested against the monomer-, dimer- and trimer-HSA conjugate, respectively, presenting different linker and protein (see Supporting Information File 1). B) Comparison of anti trimer-HSA antibody levels in sera from conjugates **14**–**17**. Each point represents individual mouse sera; horizontal bars indicate geometric mean titers (GMT) of each group with 95% statistical confidence intervals indicated by upper and lower bars.

Since the trimer **3**, among the set of conjugated synthetic oligomers, was the only structure capable of inducing IgG antibodies against the MenX CPS, we interrogated whether the sera from the three conjugated synthetic fragments were capable to recognize their own structures. To answer this question, conjugates with HSA were prepared with a similar protocol to that used for the formation of the CRM_197_ conjugates, except that a bis-succinimidyl ester penta-ethylene glycol (BS(PEG)_5_) linker was used to rule out the interference of the spacer (details are reported in Supporting Information File 1). ELISA analysis using HSA conjugates as coating reagent demonstrated that the conjugated monomer **1** and the dimer **2** at the present dose elicited extremely low levels of antibodies against themselves and were, therefore, scarcely antigenic. By contrast, the trimer **3** was the only fragment which evoked a robust antibody production against its own structure (Figure 3A).

Surprisingly, when the trimer-HSA conjugate was used as coating reagent for the ELISA analysis (Figure 3B), sera elicited by the monomer **1** exhibited low anti-trimer antibodies, whereas sera induced by the conjugated dimer **2** and the trimer **3** possessed comparable levels of antibodies against the trisaccharide structure. The IgG levels raised by the trimer-CRM_197_ conjugate **16** were in turn significantly lower (p 0.0005) than those elicited by the MenXDP15-CRM_197_ conjugate **17**, where the trisaccharide is repeated multiple times. We hypothesize that the improved binding of antibodies induced by **14** and, primarily, by **15** to the coated trisaccharide conjugate rather than to **1**- and **2**-HSA conjugates, respectively, might be the result of a better exposition of monosaccharide and disaccharide MenX units in the context of the trimer-HSA molecule, due presumably to conformational or spatial factors. However, this peculiar recognition was not observed when the native MenX CPS, which is the structure that best resembles the bacterial sugar surface and consequently of major interest for vaccine development, was used as coating reagent (Figure 2). Together, these evidences indicated that the trimer is the minimal antigenic portion of the polymer which is also capable of inducing antibodies recognizing epitopes of MenX CPS.

Next, the functionality of the induced antibodies was assessed by measuring the rabbit complement-mediated lysis of *N. meningitidis* [39]. This in vitro bactericidal assay is typically used to predict the effectiveness of anti-meningococcal vaccines [40–41]. Rabbit complement serum bactericidal assay (rSBA) titers were determined using pooled sera from mice immunized with the conjugated monomer **14**, dimer **15**, trimer **16** and MenXDP15 **17**. As it can be seen from Table 2, rSBA titers showed a trend similar to the measured ELISA IgG levels.

**Table 2:** rSBA titers of sera from immunized mice.

Sample	Antigen dose	Adjuvant	rSBA titer

PBS	-	Al phosphate	<16
**14**	0.3 µg	Al phosphate	<16
**15**	0.3 µg	Al phosphate	<16
**16**	0.3 µg	Al phosphate	512
**16**	1 µg	Al phosphate	512
MenXDP15-CRM_197 _**17**	0.3 µg	Al phosphate	8192
MenXDP15-CRM_197_ **17**	1 µg	Al phosphate	8192

Only pooled sera from mice immunized with the conjugated trimer **16** demonstrated to be functional, whereas compounds **14** and **15** did not exhibit any bactericidal activity. However, the rSBA titers of the trimer at 0.3 and 1 μg saccharide base doses were significantly lower (<10 fold in terms of titer) than MenXDP15-CRM_197_ conjugate **17** at the corresponding doses (512 and 8192, respectively).

To sum up, following conjugation to the carrier protein the chain of three repeating units constitutes the minimal antigenic structure of MenX polysaccharide, but its immunogenicity towards the native polysaccharide is lower than a medium size MenX CPS fragment (avDP 15) both in terms of IgG levels and functionality of the induced antibodies.

## Conclusion

Serogroup X *N. meningitidis* is dramatically emerging among the causative agents of meningitis, particularly in Africa. Similarly to other serogroups, polysaccharide-based conjugates have been recently proposed as target molecules for the development of a vaccine with a broader coverage. We have undertaken the preparation of neo-glycoconjugates from fully synthetic MenX fragments in order to gain structural insights about minimal epitopes of this polysaccharide, and investigate the possibility of using synthetic pure and well-defined carbohydrates for the development of a glycoconjugate vaccine.

While, at the present dose and carbohydrate loading, conjugates of the synthetic MenX PS monomer and dimer with CRM_197_ were poorly antigenic, the conjugated trimer resulted in the minimal structure eliciting antibodies that can recognize both itself and epitopes of the native polysaccharide. Furthermore, these antibodies possess anti-meningococcal bactericidal activity towards serogroup X, although in less extent than a medium size native MenX CPS fragment (avDP 15).

This finding suggests that oligomers longer than three repeating units might be required to fully mimic the polysaccharide activity, and paves the ground for a deeper understanding of the structural requirements needed to develop a conjugate vaccine based on well-defined oligosaccharides attained by chemical synthesis. The preparation of these longer chain MenX fragments, based on the synthetic improvements and also the benefits of the continuous-flow microreactor technology herein described, is currently ongoing in our laboratory and it will be reported in due course. Besides the length of the carbohydrate haptens, the saccharide loading onto the protein is another key parameter which has been shown to deeply affect the immunogenicity of glycoconjugate vaccines [42–43]. In the present preliminary study, glycoconjugates with moderate loading were compared. It has been reported for other bacterial systems that the immunogenicity of short oligosaccharides can be enhanced by increasing the number of glycan antigens incorporated onto the carrier protein. The utilization of other conjugation chemistries enabling the achievement of higher carbohydrate loadings and the study of the effect of different glycan–protein ratios on glycoconjugates prepared from these short synthetic MenX fragments also deserve further exploration.

## Experimental

**General procedure for conjugation of fragments 1–3.** This procedure is similar to that reported in reference [27]. The glycan (10 μmol) dissolved in DMSO (250 μL) containing triethylamine (25 equiv), was slowly dropped into a mixture of bis(*N*-succinimidyl) adipate (10 equiv) in DMSO (250 μL). After 3 hours under vigorous stirring, the activated oligosaccharide was purified by precipitation of the reaction mixture in nine volumes (9 mL) of ethyl acetate. The pellet obtained by subsequent centrifugation was washed with ethyl acetate (10 times × 3 mL), and freeze dried. After spectrophotometric determination of active ester groups, the pellet was incubated overnight with the protein in 100 mM NaP_i_ pH 7.2.

The glycoconjugate was washed on a 30 kDa Amicon centrifugal filter with 10 mM NaP_i_ pH 7 (8 × 100 μL), and subsequently reconstituted with 10 mM NaP_i_ pH 7. Yields (recovered glycoprotein as determined by microBCA, Pierce Thermo): 85–95%. Loading of glycoconjugate was determined by matrix-assisted laser desorption ionization time-of-flight mass spectrometry (MALDI–TOF MS; UltraFlex III MALDI–TOF/TOF instrument, Bruker Daltonics) in linear mode and with positive ion detection. The samples for analysis were prepared by mixing 2.5 μL of product and 2.5 μL of sinapinic acid matrix (Bruker Daltonics); 2.5 μL of each mixture was deposited on a samples plate, dried at room temperature for 10 min, and subjected to the spectrometer.

For SDS page analysis, the samples (5 μg) were electrophoresed on a 7% TrisAcetate gel or 4–12% Bis-Tris gel (NuPage, Invitrogen) and stained with Coomassie blue.

**Immunization of mice.** Animal experimental guidelines set forth by the Novartis Animal Care Department were followed in the conduct of all animal studies. For the formulation of the vaccines, to a volume of glycoconjugate corresponding to 0.3 μg or 1 μg/dose) aluminium phosphate (100 μl of a solution 1.2 mg/mL, 120 μg/dose) was added. The final volume of the formulation was diluted to 200 μL/dose by addition of PBS pH 7.2 buffer. An injection volume of 200 μL per mouse was used. MenX vaccines were administered to mice in 0.3 or 1 μg per dose based on saccharide content. As in [27] mice were immunized subcutaneously at day 1, 14 and 28. Bleedings were performed at day 0 (pre immune), day 28 (post 2) and day 42 (post 3). Control groups received PBS with adjuvant.

**ELISA analysis.** The antibody response induced by the glycoconjugates against the MenX polysaccharide and the HSA conjugates (see Supporting Information File 1) were measured by ELISA. Similarly as described in [27], plates were coated with the polysaccharide by adding 100 μL/well of a 5 μg/mL polysaccharide solution in pH 8.2 PBS buffer, and with the HSA conjugates adding 100 μL/well of a 2 μg/mL in term of protein solution in pH 7.2 PBS buffer, followed by incubation overnight at 4 °C. Coating solutions were removed from the plates by washing each well three times with PBS buffer containing 0.05% of Tween 20 (Sigma) (TPBS). A blocking step was performed by adding 100 μL of BSA solution at 3% in TPBS and incubating the plates 1 h at 37 °C. Blocking solution was removed from the plates by washing three times per well with TPBS. 200 μL of pre-diluted serum (1:25 for pre immune, 1:200 for a reference serum, 1:50–1:100 for test sera) was added to the first well of each column of the plate, while 100 μL of TPBS was dispensed into the remaining wells. Eight two-fold serial dilutions along each column were then performed by transferring from well to well 100 μL of sera solutions. After primary Abs dilution, plates were incubated for 2 h at 37 °C. After three washings with TPBS, 100 μL TPBS solutions of secondary antibody alkaline phosphatases conjugates (anti-mouse IgG 1:10000, anti-mouse IgM 1:5000 Sigma-Aldrich) were added and the plates incubated 1 h at 37 °C. Three more washes with TPBS were performed, when 100 μL/well of a 1 mg/mL of p-NPP (Sigma) in a 0.5 M diethanolamine buffer pH 9.6 were added. After 30 min of incubation at room temperature, plates were read at 405 nm using a Biorad plate reader. Raw data acquisition was performed by Microplate Manager Software (Biorad). Sera titers were expressed as the reciprocal of sera dilution corresponding to a cut-off OD = 1. Each immunization group is represented as the geometrical mean (GMT) of the single mouse titers. The statistical and graphical analysis was performed by GraphPad 5.0 software.

**Rabbit serum bactericidal assay (rSBA).** The functionality of antibodies induced by vaccine immunization was assessed by measuring the complement-mediated lysis of *N. meningitidis* with an in vitro bactericidal assay as described in the literature [15,26]. Titers were expressed as the reciprocal serum dilution resulting in 50% of bactericidal killing. Z9615 (MenX) was used as reference strain.

## Supporting Information

File 1 Experimental procedures for the synthesis of compounds **1**, **5**, **6**, **7**, **9**, **19**, copies of ^1^H NMR and ^13^C NMR spectra of compounds **5–6** and ^1^H NMR, ^13^C NMR and ^31^P NMR spectra of compounds **1**, **7**, **9**.
