# Supplementary material for: Synthesis and immunological evaluation of protein conjugates of Neisseria meningitidis X capsular polysaccharide fragments
Source: Beilstein J Org Chem. 2014 Oct 13;10:2367–76. doi: 10.3762/bjoc.10.247 (PMC4222376; doi:10.3762/bjoc.10.247)

Supporting Information

for

Synthesis and immunological evaluation of protein conjugates of *Neisseria meningitidis* X capsular polysaccharide fragments

Laura Morelli[1], Damiano Cancogni[1], Marta Tontini[2], Alberto Nilo[2], Sara Filippini[2], Paolo Costantino[2], Maria Rosaria Romano[2], Francesco Berti[2], Roberto Adamo*[2] and Luigi Lay*[1]

Address: [1]Dipartimento di Chimica and ISTM-CNR, Università degli Studi di Milano, via Golgi 19, I-20133 Milano, Italy and [2]Novartis Vaccines, Via Fiorentina 1, 53100 Siena, Italy

Email: Luigi Lay - luigi.lay@unimi.it; Roberto Adamo - roberto.adamo@novartis.com

Experimental procedures for the synthesis of compounds 1, 5, 6, 7, 9, 19, copies of ^{1}H NMR and ^{13}C NMR spectra of compounds 5–6 and ^{1}H NMR, ^{13}C NMR and ^{31}P NMR spectra of compounds 1, 7, 9

1. General methods S2

2. Conjugation to CRM_{197} S7

3. Anti MenX CPS IgM levels S8

4. Conjugation to HSA S9

5. ^{1}H NMR, ^{13}C NMR and ^{31}P NMR of the synthesized compounds S12

1. General methods

The reactions performed under microfluidic conditions were carried out in a glass microreactor with an internal volume of 100 μL purchased by Future Chemistry®. The reagent solutions were pumped into the microreactor using a KDS two syringes nanoliter pump, series 101 purchased by Sigma-Aldrich®, and enabling the use of solvent volumes up to 5 mL. All commercially available reagents including dry solvents were used as received. Nonvolatile materials were dried under high vacuum. Reactions were monitored by thin-layer chromatography on pre-coated Merck silica gel 60 F254 plates and visualized by staining with a solution of cerium sulfate (1g) and ammonium heptamolybdate tetrahydrate (27 g) in water (469 mL) and concentrated sulfuric acid (31 mL). Flash chromatography was performed on Fluka silica gel 60. All chemical yields are intended for isolated yields after chromatographic purification. NMR spectra were recorded at 300 K on spectrometer operating at 400 MHz. Proton chemical shifts are reported in ppm (δ) with the solvent reference relative to tetramethylsilane (TMS) employed as the internal standard ($CDCl_3$ δ = 7.26 ppm). *J* values are given in Hz. Carbon chemical shifts are reported in ppm (δ) relative to TMS with the respective solvent resonance as the internal standard ($CDCl_3$, δ = 77.0 ppm). Phosphorous chemical shift are reported in ppm (δ) relative to H_3PO_4. High resolution mass spectra (HRMS) were performed at CIGA (Centro Interdipartimentale Grandi Apparecchiature), with mass spectrometer APEX II & Xmass software (Bruker Daltonics). ESI-MS spectra were recorded on a JEOL AX-505 spectrometer or with micro hybrid quadrupole time of flight (Q-Tof) Mass Spectrometer (Waters) with Electronspray Ionization (ESI) source and *MassLinx NT* software.

1.1. Thexyldimethylsilyl 2-acetamido-4-*O*-acetyl-3,6-di-*O*-benzyl-2-deoxy-β-D-glucopyranoside (5)

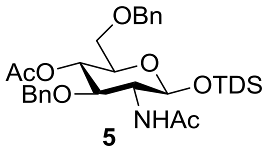

5

To a mixture of thexyldimethylsilyl 4-*O*-acetyl-2-azido-3,6-di-*O*-benzyl-2-deoxy-β-D-glucopyranoside **4** (2.00 g, 3.50 mmol) and $NiCl_2•6H_2O$ (2.50 g, 10.5 mmol) in MeOH (60 mL) $NaBH_4$ (1.06 g, 28.0 mmol) was added at 0 °C in small portion. The mixture was stirred at 0 °C and, after consumption of the starting material (monitored by TLC analysis), Ac_2O (3.30 mL, 35.0 mmol) was added. After 12 h the mixture was concentrated under reduced pressure, diluted with CH_2Cl_2 and washed three times with

water. The organic phase was dried (Na_2SO_4), concentrated, and purified by flash chromatography (Hex/EtOAc 7:3) to give the compound **5** (1.76 g, 3.01 mmol, 86% yield). $[\alpha]_D^{25}$ = -2.92 (*c* = 1.3 in MeOH); ^{1}H NMR (400 MHz, $CDCl_3$) δ (ppm) 7.37 – 7.20 (m, 10H, Ar), 5.60 (s, 1H, NH), 5.19 (d, $J_{1,2}$ = 7.8 Hz, 1H, H-1), 4.99 (t, $J_{4,5}$ = $J_{4,3}$ = 9.5 Hz, 1H, H-4), 4.59 (q, *J* = 11.5 Hz, 2H, C*H*$_2$Ph), 4.52 (s, 2H, C*H*$_2$Ph), 4.33 (dd, $J_{3,2}$ = 10.3, $J_{3,4}$ = 9.5 Hz, 1H, H-3), 3.70 – 3.62 (m, 1H, H-5), 3.54 (d, *J* = 4.5 Hz, 2H, H-6, H-6'), 3.18 (dt, $J_{2,3}$ = 10.3, $J_{1,2}$ = 7.8 Hz, 1H, H-2), 1.88 (s, 3H, CH_3CO), 1.87 (s, 3H, CH_3CO), 1.62 (ept, *J* = 7.0 Hz, 1H, CH TDS), 0.87 (d, *J* = 1.8 Hz, 3H, CH_3 TDS), 0.86 (d, *J* = 1.7 Hz, 3H, CH_3 TDS), 0.84 (d, *J* = 2.2 Hz, 6H, 2 CH_3 TDS), 0.17 (s, 3H, CH_3Si TDS), 0.13 (s, 3H, CH_3Si TDS); ^{13}C NMR (101 MHz, $CDCl_3$) δ (ppm) 170.6, 169.7 (CO), 138.5, 138.3 (C_q Ar), 128.9, 128.8, 128.6, 128.5, 128.0, 127.8, 127.7 (CH Ar), 94.6 (C-1), 78.0 (C-3), 73.9 (CH_2Ph), 73.6 (CH_2Ph), 73.4 (C-5), 72.2 (C-4), 70.2 (C-6), 60.2 (C-2), 34.2 (CH TDS), 24.9 (C_q TDS), 23.6 (CH_3CO), 21.0, 20.2, 18.7 (CH_3 TDS), -1.7, -3.4 (CH_3Si TDS); ESI-HRMS $[M-Na]^+$ *m/z* calc for $C_{32}H_{47}NO_7SiNa$ 608.3019, found 608.2830

1.2. 2-Acetamido-4-*O*-acetyl-3,6-di-*O*-benzyl-2-deoxy-α-D-glucopyranose (6)

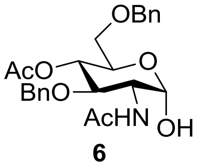

6

To a solution of **5** (1.30 g, 2.20 mmol) in anhydrous THF (20 mL) tetrabutylammonium fluoride 1M in THF (3.30 mL, 3.30 mmol) was added at -40°C. After stirring for 5 h the reaction was complete, and the mixture was warmed up to r.t., poured in DCM, and washed three times with brine. The organic layer was dried (Na_2SO_4), filtered and concentrated. The residue was purified by column chromatography (Hex/EtOAc 2:8) to give compound **6** (0.79 g, 1.78 mmol, 81% yield). $[\alpha]_D^{25}$ = +38.46 (*c* = 1.1 in MeOH); ^{1}H NMR (400 MHz, $CDCl_3$) δ (ppm) 7.36 – 7.23 (m, 10H, Ar), 5.49 (d, *J* = 8.7 Hz, 1H, NH), 5.23 (d, $J_{1,2}$ = 3.5 Hz, 1H, H-1), 5.07 (t, $J_{4,5}$ = $J_{4,3}$ = 9.5 Hz, 1H, H-4), 4.63 (d, *J* = 11.5 Hz, 1H, ½ C*H*$_2$Ph), 4.52 (d, *J* = 11.5 Hz, 1H, ½ C*H*$_2$Ph), 4.51 (s, 2H, C*H*$_2$Ph), 4.23 – 4.05 (m, 2H, H-2, H-5), 3.86 (dd, $J_{3,2}$ = 10.5, $J_{3,4}$ = 9.5 Hz, 1H, H-3), 3.53 – 3.45 (m, 2H, H-6, H-6'), 1.94 (s, 3H, CH_3CO), 1.85 (s, 3H, CH_3CO); ^{13}C NMR (101 MHz, $CDCl_3$) δ (ppm) 170.5, 169.7 (CO), 138.2, 137.8 (C_q Ar), 128.7, 128.5, 128.2, 128.2, 128.1, 128.0, 127.9 (CH Ar), 92.0 (C-1), 76.8 (C-3), 73.7 (CH_2Ph), 73.0 (CH_2Ph), 71.2 (C-4), 69.6 (C-6), 69.5 (C-5), 53.0 (C-2), 23.5 (CH_3CO), 21.0 (CH_3CO); ESI-HRMS $[M-Na]^+$ *m/z* calc for $C_{24}H_{29}NO_7Na$ 466.1842, found 466.1653.

1.3. 2-Acetamido-4-*O*-acetyl-3,6-di-*O*-benzyl-2-deoxy-α-D-glucopyranosyl hydrogenphosphonate triethylammonium salt (7)

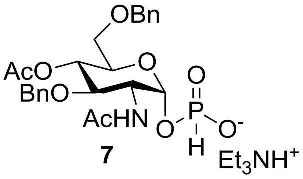

7

Batch procedure: Salicylchlorophosphite (96 mg, 0.473 mmol) was slowly added to a solution of alcohol **6** (140 mg, 0.315 mmol) in dry pyridine (1 mL) at 0°C. The reaction was stirred at r.t. for 3.5 h. Then a 1 M solution of TEAB (4 mL/mmol) was added to the reaction at r.t., and the mixture was diluted with CH_2Cl_2, washed three times with cold TEAB (0.5 M), dried (Na_2SO_4), filtered and concentrated. The crude was purified by flash chromatography (CH_2Cl_2/MeOH 9:1 + 1% TEA). The H-phosphonate was dissolved in $CHCl_3$ and was stabilized by washing with 0.25 M cold TEAB, then dried (Na_2SO_4), filtered and concentrated to dryness giving product **7** (119 mg, 0.195 mmol, 62% yield).

Flow procedure: A solution of alcohol **6** (89 mg, 0.1 M) pyridine (2 mL) was prepared (solution A). A solution of salycil chlorophosphite (81 mg, 0.2 M) in CH_3CN (2 mL) was prepared in a separated flask (solution B). Equal volumes (1 mL) of the two solutions were taken and injected into the microreactor (internal volume = 100 μL) via a double syringe pump, setting the flow rate at 16.5 μL/min (total flow rate = 33 μL/min, corresponding to a residence time = 3 min). The mixture was flown for a total time of 0.5 h from the microreactor to a 1 M TEAB aqueous solution (1 mL) in order to quench the reaction. The reaction mixture was concentrated *in vacuo* and the crude was purified by flash chromatography (CH_2Cl_2/MeOH 9:1 + 1% TEA). The H-phosphonate was dissolved in $CHCl_3$ and was stabilized by washing with 0.25 M cold TEAB, then dried (Na_2SO_4), filtered and concentrated to dryness giving product **7** as a white solid (46 mg, 76%). ^{1}H NMR (400 MHz, $CDCl_3$) δ (ppm) 7.33 – 7.15 (m, 10H, Ar), 6.93 (d, J = 631.8 Hz, 1H, *H*-P), 6.41 (d, J = 8.8 Hz, 1H, N*H*), 5.53 (dd, $J_{1,P}$ = 8.4, $J_{1,2}$ = 3.2 Hz, 1H, H-1), 5.11 (t, $J_{4,3}$ = $J_{4,5}$ = 9.7 Hz, 1H, H-4), 4. 57 (s, 1H, ½ C*H*$_2$Ph), 4.56 (s, 1H, ½ CH_2Ph), 4.50 – 4.40 (m, 2H, C*H*$_2$Ph), 4.37 – 4.26 (m, 1H, H-2), 4.19 – 4.11 (m, 1H, H-5), 3.85 (t, $J_{3,4}$ = 9.7 Hz, 1H, H-3), 3.50 – 3.43 (m, 2H, H-6, H-6'), 1.86 (s, 6H, C*H*$_3$CO); ^{31}P NMR (162 MHz, $CDCl_3$) δ (ppm) 1.87; ^{13}C NMR (101 MHz, $CDCl_3$) δ (ppm) 170.2, 169.7 (CO), 138.5, 138.1 (C_q Ar), 128.4, 128.4, 123.0, 127.7 (CH Ar), 93.5, 93.3 (C-1), 78.0 (C-3), 73.5 (*C*H$_2$Ph), 73.4 (*C*H$_2$Ph), 71.0 (C-4), 70.4 (C-5), 69.7 (C-6), 52.4, 52.4 (C-2), 23.4 (*C*H$_3$CO), 20.9 (*C*H$_3$CO).

1.4. 3-(*N*-Carbobenzyloxy)aminopropyl 1-*O*-2-acetamido-4-*O*-acetyl-3,6-di-*O*-benzyl-2-deoxy-α-D-glucopyranosyl phosphate, triethylammonium salt (9)

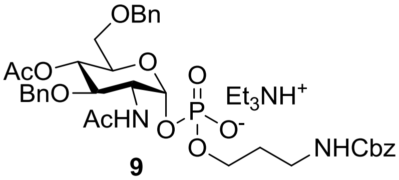

9

The H-phosphonate **8** (0.32 g, 0.85 mmol) and the hemiacetal **6** (0.31 g, 0.69 mmol) were first co-evaporated three times with dry toluene, thereafter they are dried by high vacuum pump overnight. The reactants were dissolved in pyridine (7 mL), then pivaloyl chloride (0.26 mL, 2.13 mmol) was added drop-wise at 0°C and the mixture was warmed up to r.t. After 2 h, the reaction completion was assessed by TLC. The mixture was cooled to -40°C, and a freshly prepared 0.5 M solution of iodine (0.54 g, 2.13 mmol) in pyridine/water 19:1 was added. The oxidation was completed in 3 h at 0°C and quenched by dropwise addition of a 0.5 M solution of $Na_2S_2O_3 \cdot 5\ H_2O$ (10% w/v). The mixture was diluted with $CHCl_3$, washed two times with $Na_2S_2O_3 \cdot 5H_2O$ (0.5 M), then with cold TEAB (0.5 M), dried (Na_2SO_4), filtered and concentrated. The crude was purified by flash chromatography (DCM/MeOH 8:2 + 1% TEA). The phosphodiester was dissolved in $CHCl_3$ and was stabilized by washing with 0.25 M cold TEAB, then dried (Na_2SO_4), filtered and concentrated to dryness to provide product **9** (0.253 g, 0.31 mmol, 45% yield). $[\alpha]_D^{25}$ = +39.47 (*c* = 1.4 in $CHCl_3$); ^{1}H NMR (400 MHz, $CDCl_3$) δ (ppm) 7.34 – 7.18 (m, 15H, Ar), 6.79 (d, *J* = 8.5 Hz, 1H, N*H*Ac), 5.95 (t, *J* = 5.4 Hz, 1H, N*H*Cbz), 5.49 (dd, $J_{1,P}$ = 7.5, $J_{1,2}$ = 3.5 Hz, 1H, H-1), 5.12 – 4.99 (m, 3H, H-4, CH_2Ph), 4.58 (s, 2H, CH_2Ph), 4.47 – 4.37 (m, 2H, CH_2Ph), 4.36 – 4.27 (m, 1H, H-2), 4.18 – 4.08 (m, 1H, H-5), 4.00 – 3.90 (m, 2H, CH_2O linker), 3.85 (t, $J_{3,4}$ = $J_{3,2}$ = 9.9 Hz, 1H, H-3), 3.52 – 3.39 (m, 2H, H-6, H-6'), 3.30 – 3.18 (m, 2H, CH_2NH), 1.87 (s, 3H, CH_3CO), 1.86 (s, 3H, CH_3CO), 1.65 (m, 2H, CH_2 linker); ^{31}P NMR (162 MHz, $CDCl_3$) δ -0.43; ^{13}C NMR (101 MHz, $CDCl_3$) δ 170.6, 169.8 (CO), 156.7 (C_q Ar-NH), 138.5, 137.9, 136.9 (C_q Ar), 128.6, 128.4, 128.4, 128.1, 128.1, 128.0, 127.8, 127.7 (CH Ar), 94.7 (C-1), 77.6 (C-3), 73. 6 (CH_2Ph), 73.4 (CH_2Ph), 71.0 (C-4), 70.5 (C-5), 69.8 (C-6), 66.5 (CH_2Ph), 63.2 (*C*H_2O linker), 52.7 (C-2), 37.5 (*C*H_2NH), 30.5 (*C*H_2 linker); ESI-HRMS $[M\text{-}Et_3NH^+]^-$ *m/z* calc for $C_{35}H_{42}N_2O_{12}P^-$ 713.24754, found 713.24653.

1.5. 3-Aminopropyl 1-*O*-(2-acetamido-2-deoxy-α-D-glucopyranosyl phosphate), sodium salt (1)

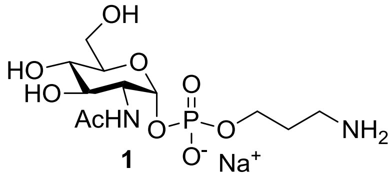

Compound **9** (60.3 mg, 0.075 mmol) was dissolved in MeOH (1 mL) and a solution of NaOMe 0.1 M in MeOH (0.08 mL) was added. The reaction was stirred at room temperature and monitored by TLC analysis. After reaction completion (1 h) the solution was neutralized with Amberlite IR-120 resin (H^+ form), filtered and concentrated to dryness. ^{1}H NMR of the crude residue confirmed 4-*O*-deacetylation, and compound **10** was used in the subsequent reaction without further purification. Similarly as described in [2], compound **10** (55.3 mg, 0.073 mmol) was dissolved in a MeOH/H_2O mixture (1:1, 4 mL), then Pd/C catalyst (10%, 0.10 g) was added, and the reaction mixture was vigorously stirred under hydrogen at room temperature overnight. The reaction was diluted with H_2O, filtered over a Celite pad, concentrated under reduced pressure and finally lyophilized to give an amorphous solid. This was dissolved in H_2O and first eluted through a column filled with Dowex 50W X8 resin (H^+ form) and then through a column filled with the same resin in Na^+ form. The eluted solution was lyophilized to afford **1** as a white foam (27.4 mg, 0.072 mmol, 96%). The characterization data of compound **1** were in agreement with those previously reported.[1]

[1] L. Morelli, L. Lay, *ARKIVOC* **2013**, 166-184

[2] M. I. Torres-Sanchez, C. Zaccaria, B. Buzzi, G. Miglio, G. Lombardi, L. Polito, G. Russo, L. Lay *Chem. Eur. J.* **2007**, *13*, 6623 - 6635

2. Conjugation to CRM_{197}

2.1. MALDI–TOF spectra of CRM_{197} glycoconjugates

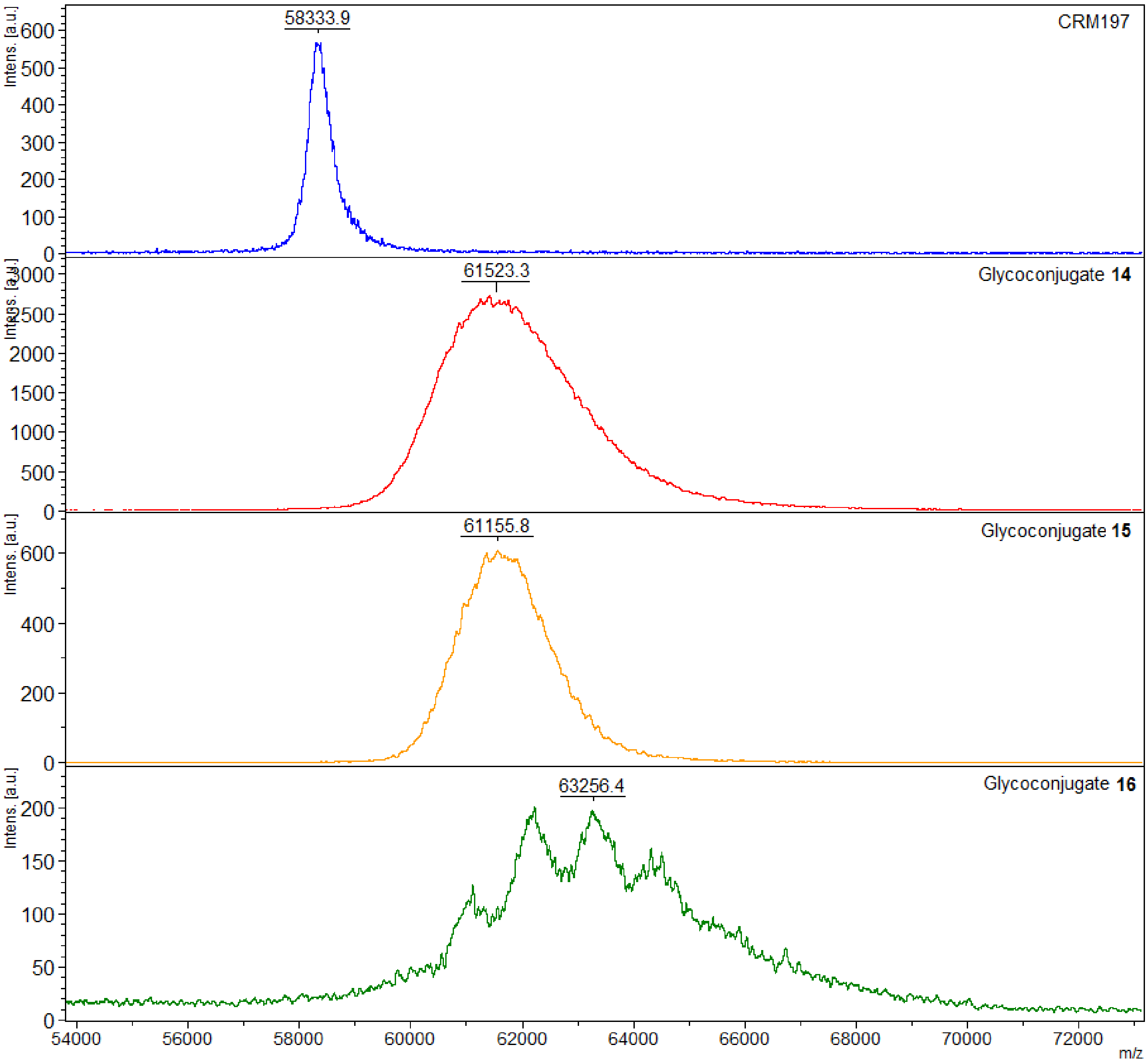

2.2. SDS page

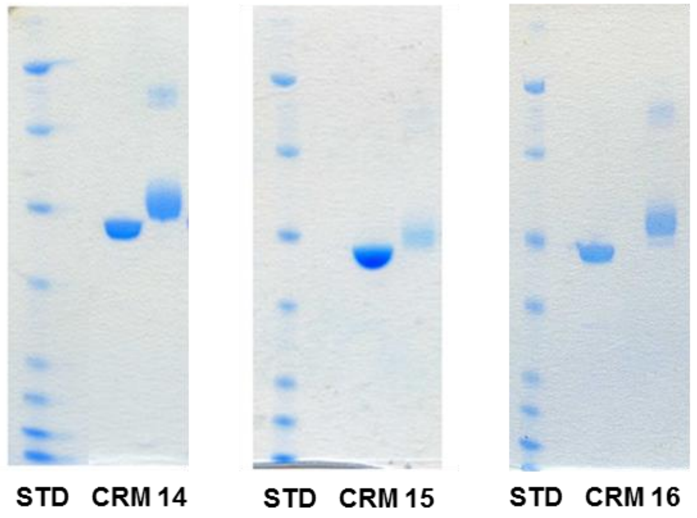

3. Anti MenX CPS IgM levels

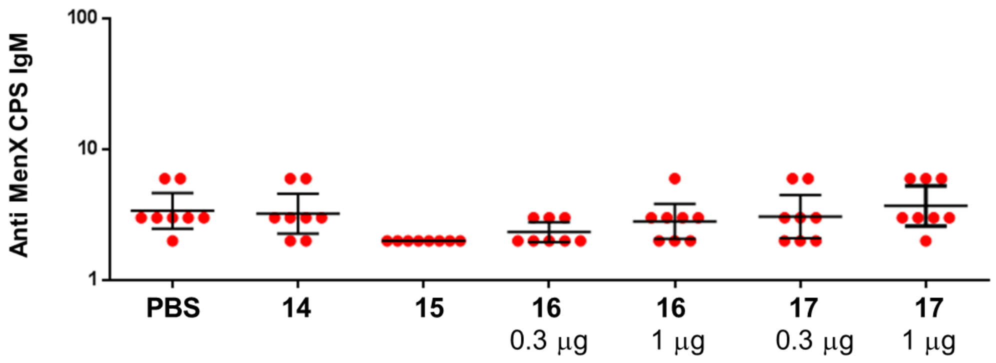

Figure S1**:** IgM levels detected at OD = 1 in individual post 3 sera (sera collected two weeks after the third immunization) of BALB/c mice immunization at 0.3 or 1 µg saccharide dose of antigen against MenX CPS as coating plate. Each dot represents individual mouse sera; horizontal bars indicate geometric mean titers (GMT) of each group with 95% statistical confidence intervals indicated by upper and lower bars. The analysis was conducted as described in the Experimental part.

3. Conjugation to HSA

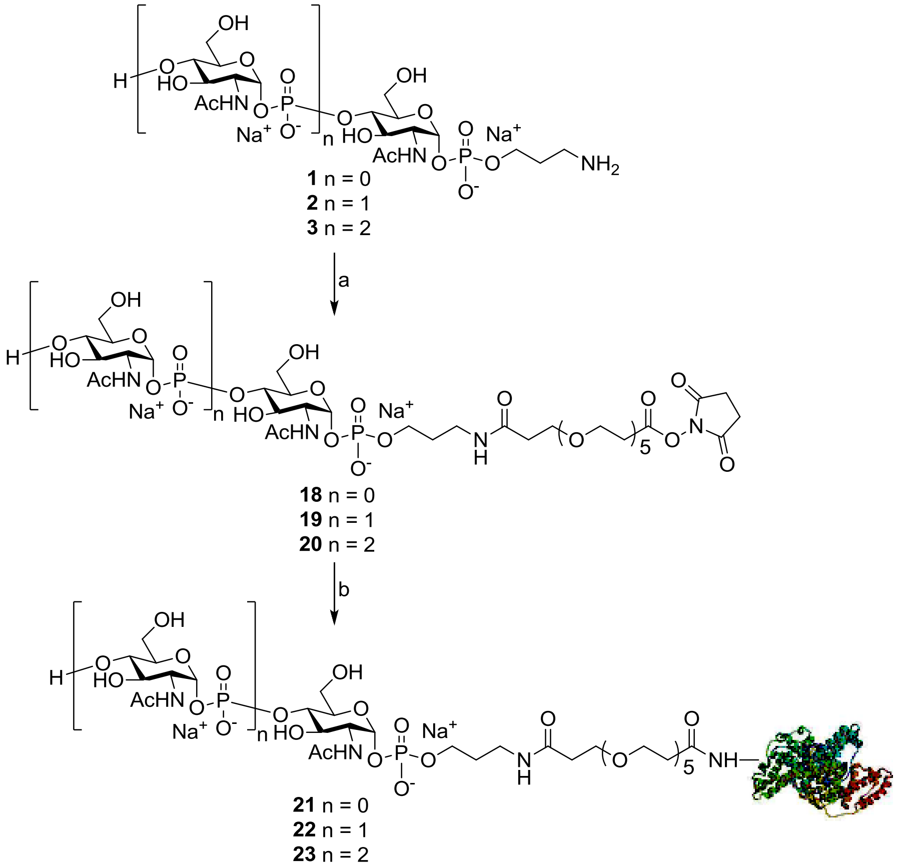

Scheme S1 (a) Bis-succinimidyl penta-ethylene glicol ester BS(PEG)$_5$, Et_3N, DMSO; (b) HSA, 100 mM NaPi, pH 7.2

Procedure for conjugation of saccharide 3 with HSA. N-hydroxydisuccinimidyl (BS) PEG$_5$ was purchased from Pierce. The sugar (10 μmol) dissolved in DMSO (250 μL) containing triethylamine (25 eq), was added drop-wise to a mixture of BS(PEG)$_5$ (10 eq) in DMSO (250 μL). After 3 hours under vigorous stirring, the activated oligosaccharide was precipitated by addition of nine volumes (9 mL) of ethyl acetate. The pellet obtained by subsequent centrifugation was washed with ethyl acetate (10 times × 3 mL), and freeze dried. After spectrophotometric determination of active ester groups, the pellet was incubated overnight with the protein in 100 mM NaPi pH 7.2 at an activated glycan:protein ratio of 50:1 for compounds **18** and **19**, and 75:1 for compound **20**.

The glycoconjugate was washed on a 30 kDa Amicon centrifugal filter with 10 mM NaPi pH 7 (8 × 100 μL), and subsequently reconstituted with 10 mM NaPi pH. By MALDI-TOF MS a loading of 3, 2 and 6 glycans/mmol of protein was determined for glycoconjugates **21**, **22** and **23**, respectively.

For SDS page analysis, the samples (5 μg) were electrophoresed on a 7% TrisAcetate gel or 4-12% Bis-Tris gel (NuPage, Invitrogen) and stained with Coomassie blue.

4.1. MALDI TOF spectra of HSA glycoconjugates

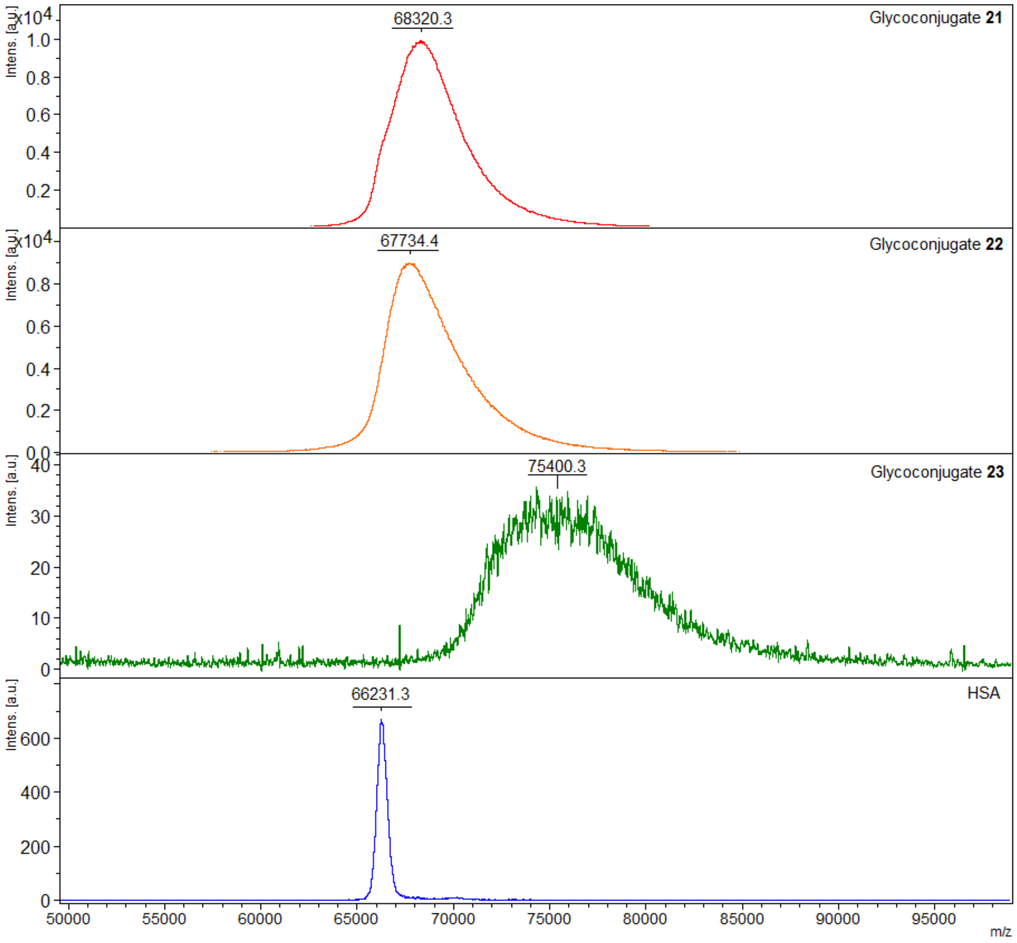

4.2. SDS Page

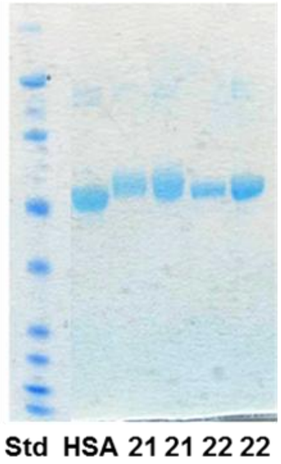

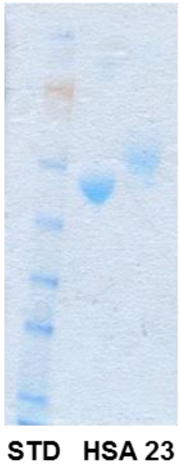

5. ^{1}H NMR, ^{13}C NMR and ^{31}P NMR of the synthesized compounds

^{1}H NMR (400 MHz, $CDCl_3$) spectrum of compound **5**

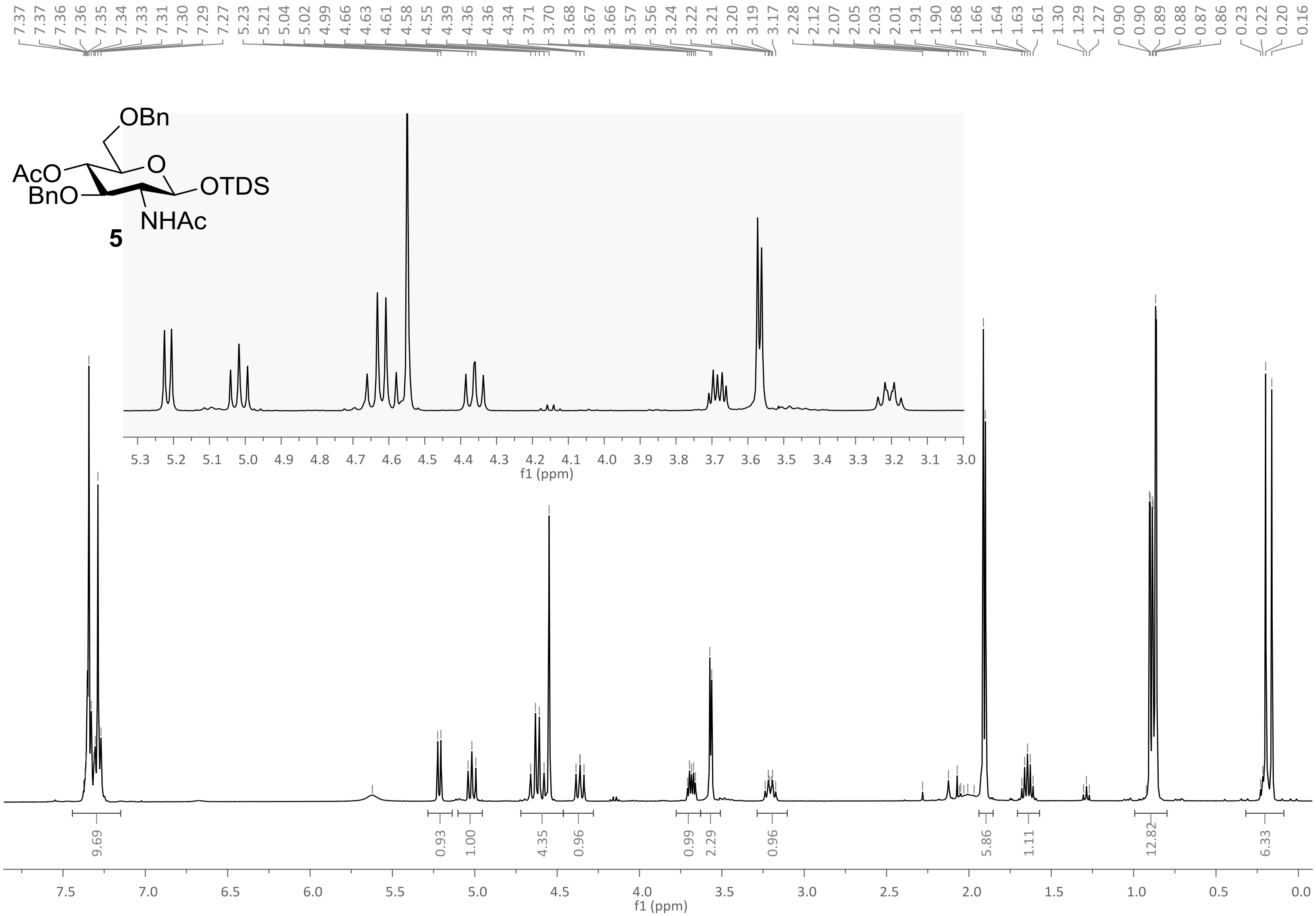

^{13}C NMR (101 MHz, $CDCl_3$) spectrum of compound **5**

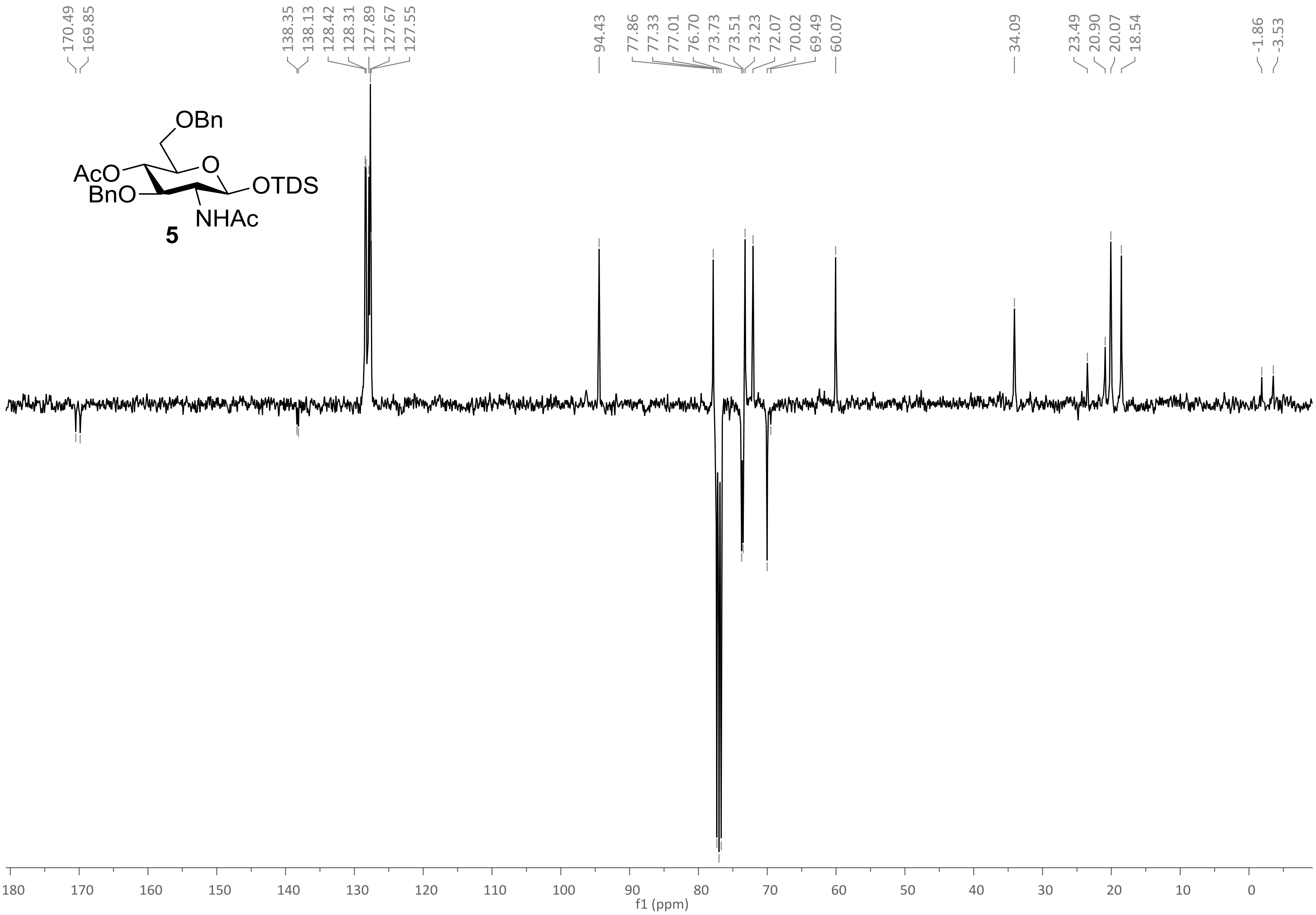

^{1}H NMR (400 MHz, $CDCl_3$) spectrum of compound **6**

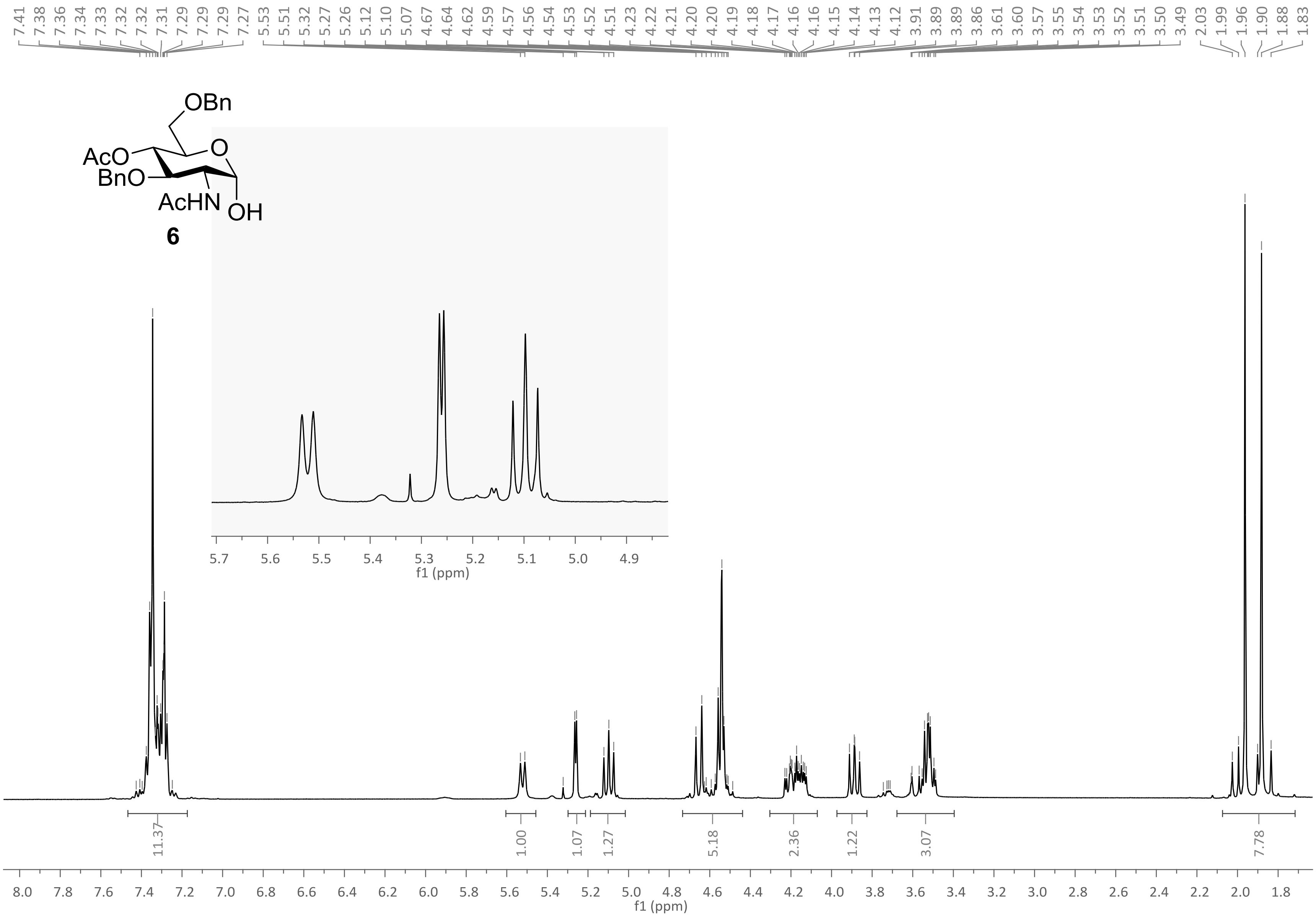

^{13}C NMR (101 MHz, $CDCl_3$) spectrum of compound **6**

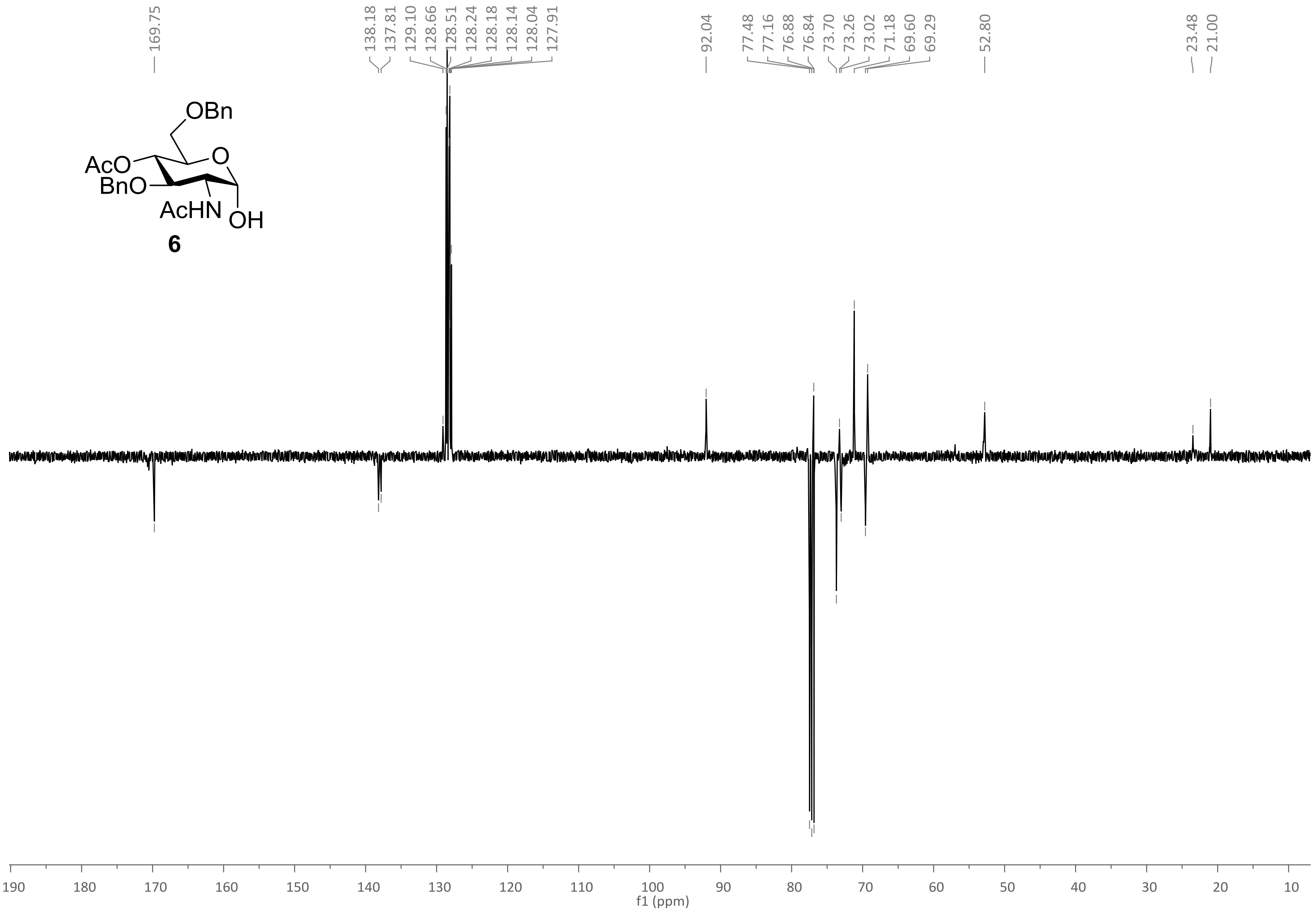

^{1}H NMR (400 MHz, $CDCl_3$) spectrum of compound **7**

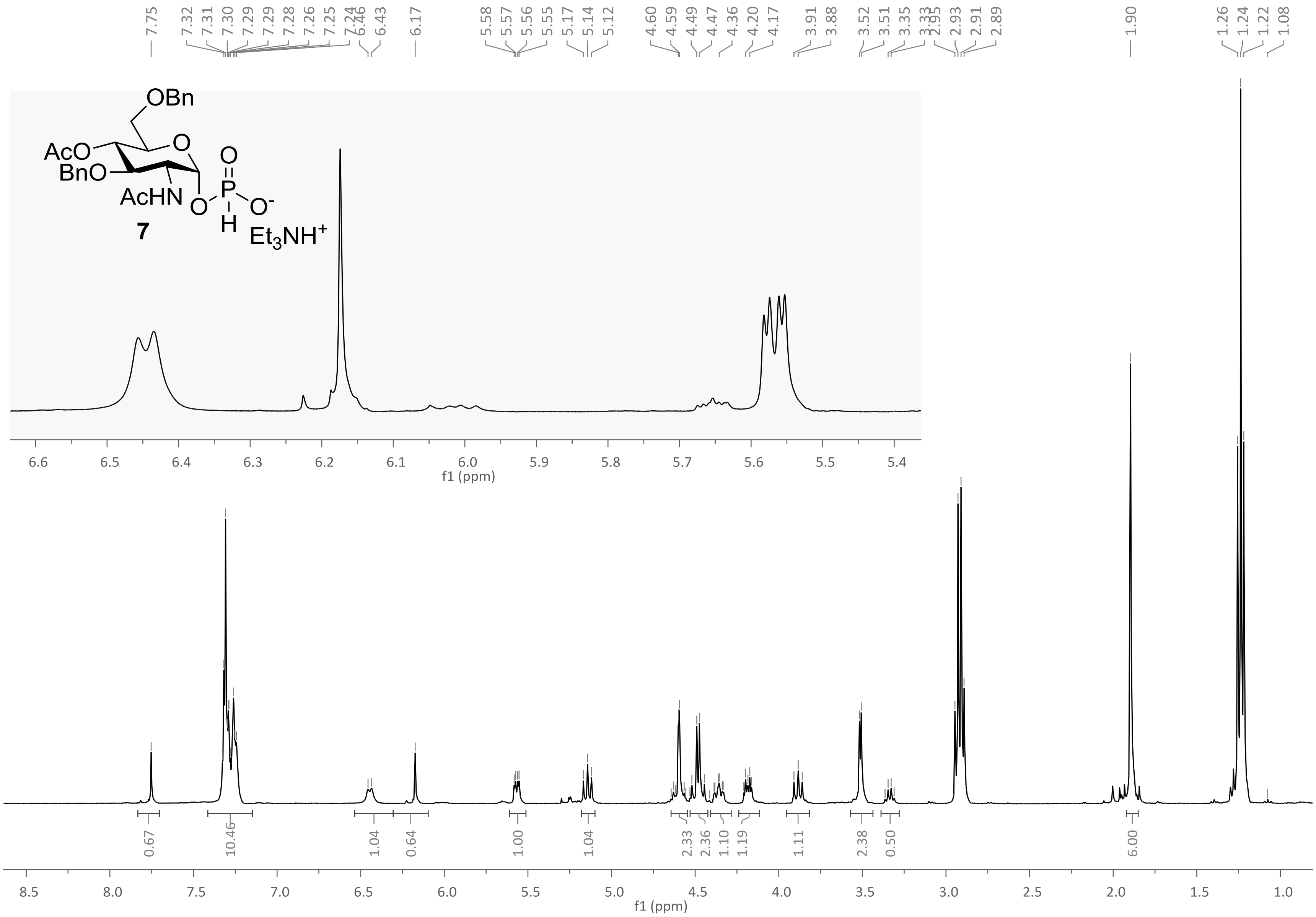

^{31}P NMR (162 MHz, $CDCl_3$) spectrum of compound **7**

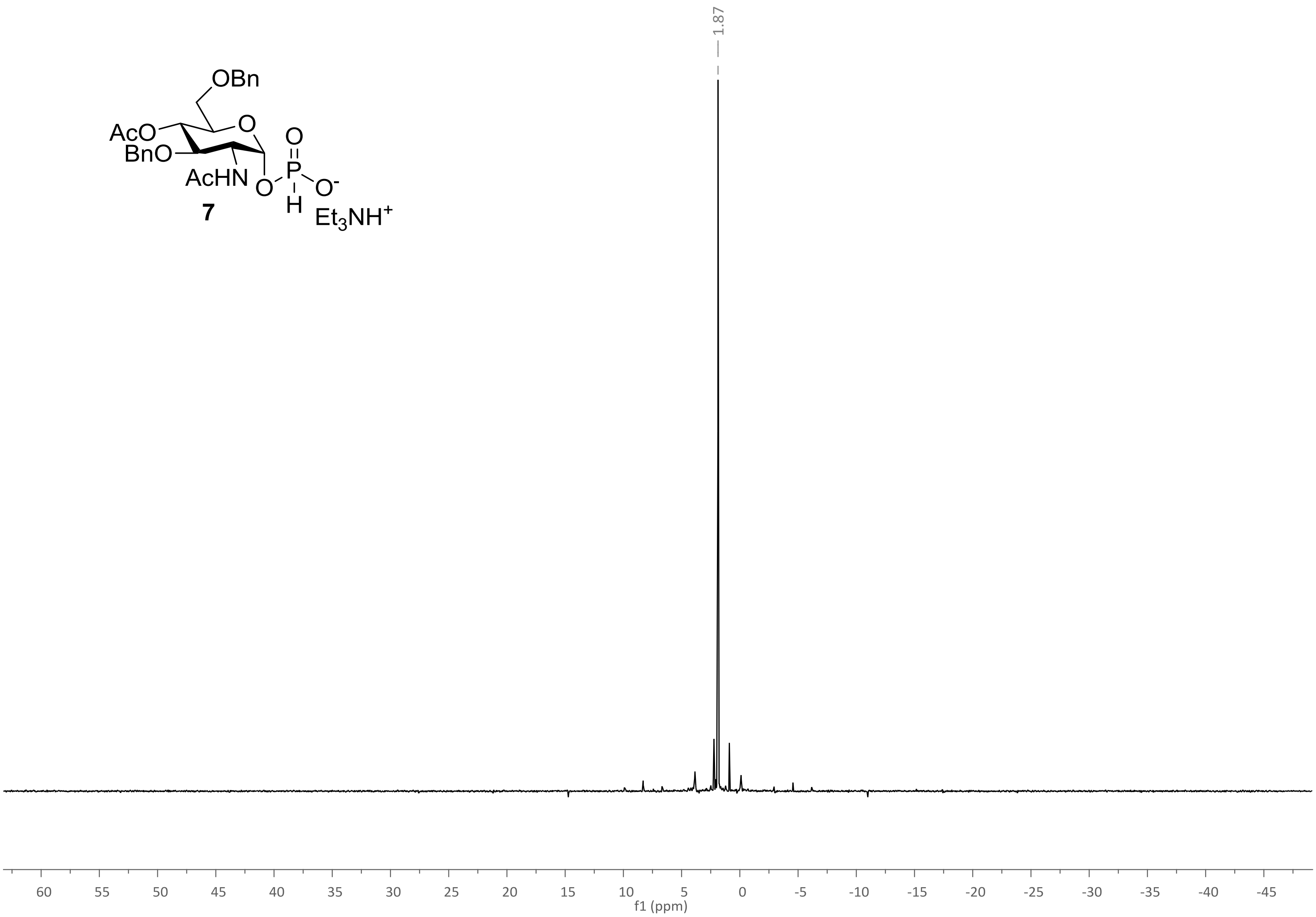

^{13}C NMR (101 MHz, $CDCl_3$) spectrum of compound **7**

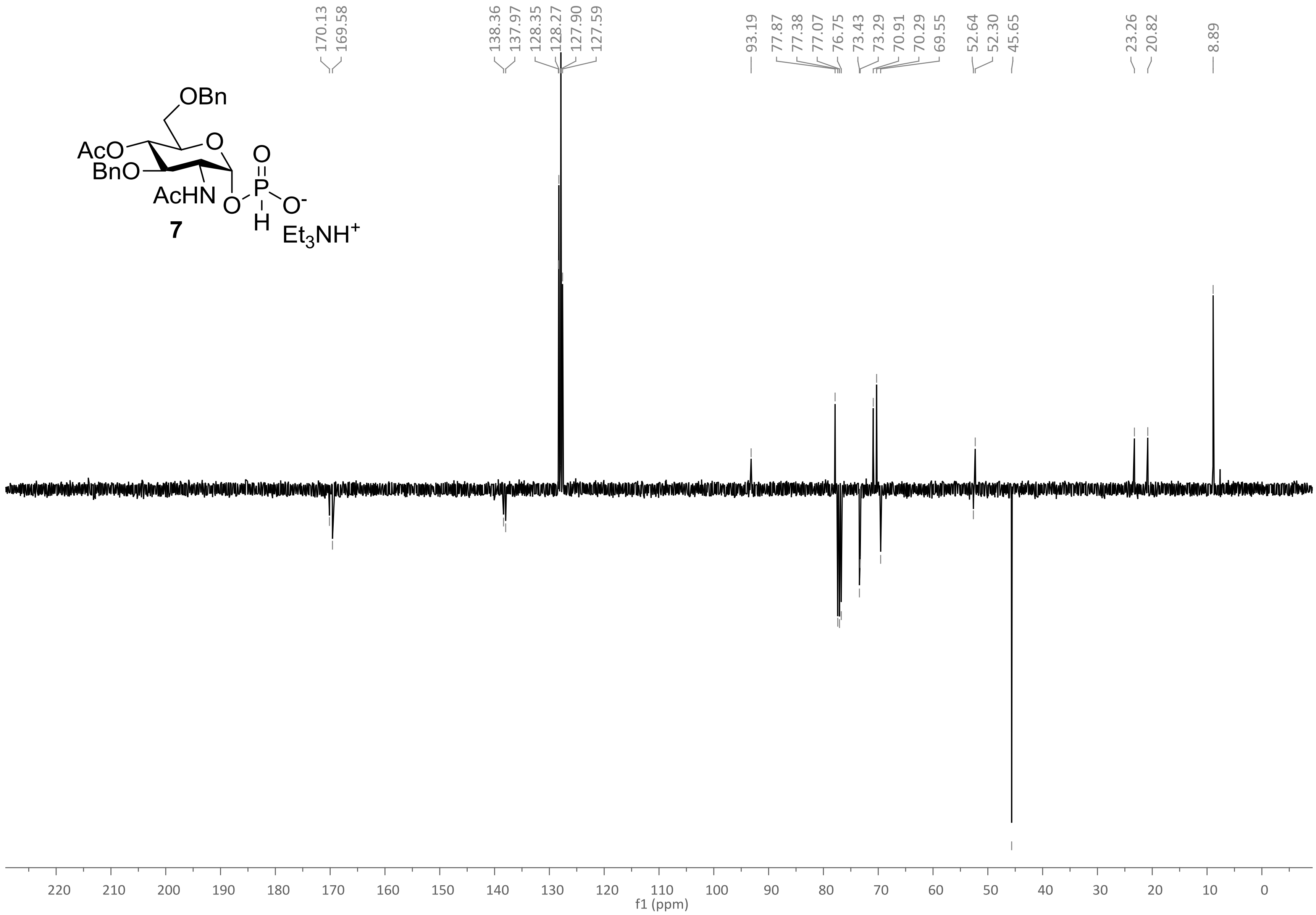

^{1}H NMR (400 MHz, $CDCl_3$) spectrum of compound **9**

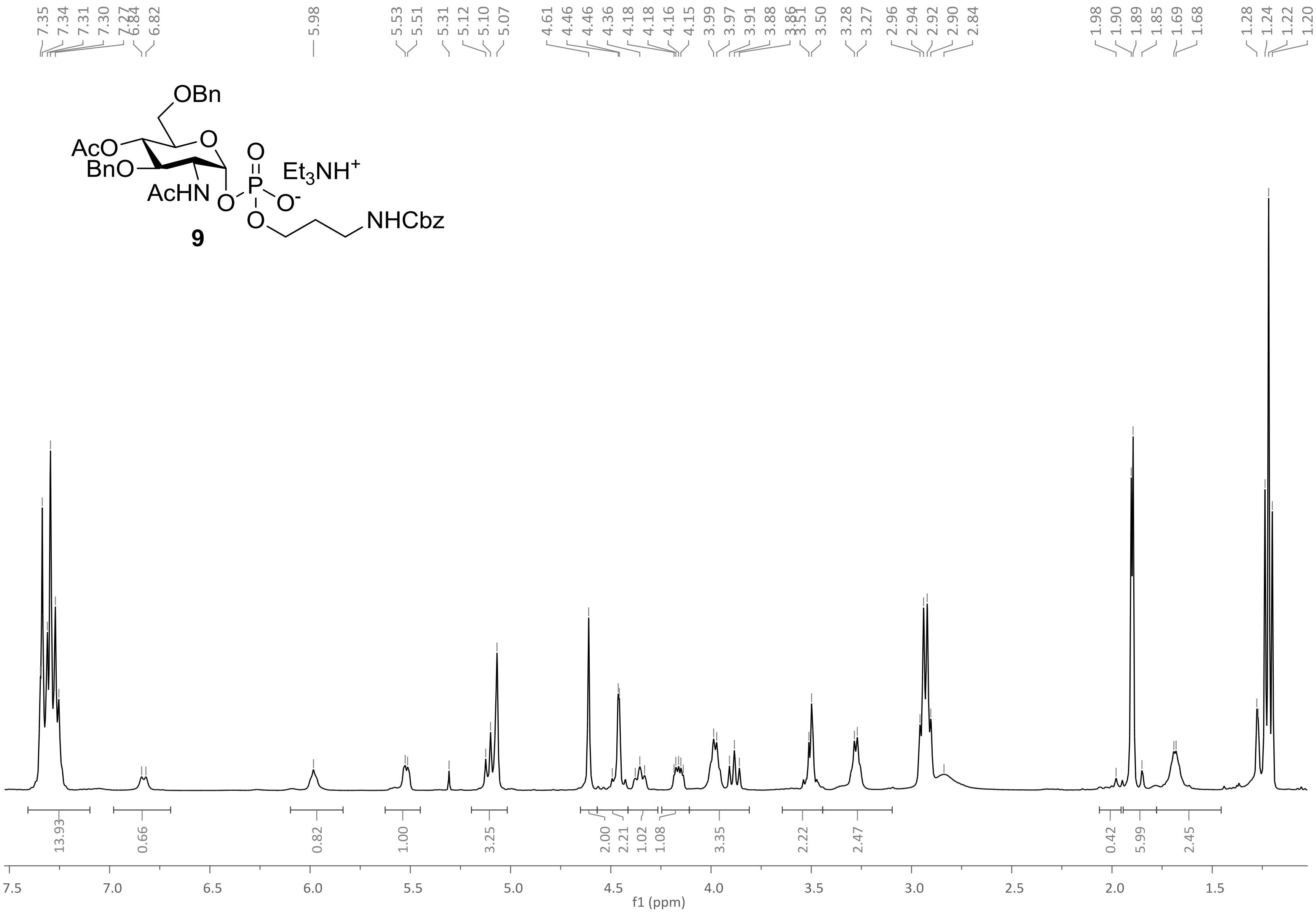

^{31}P NMR (162 MHz, $CDCl_3$) spectrum of compound **9**

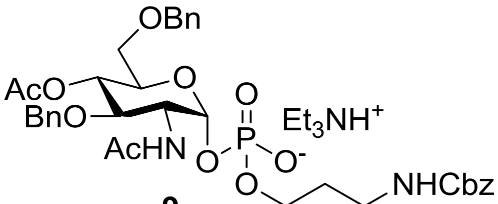

9

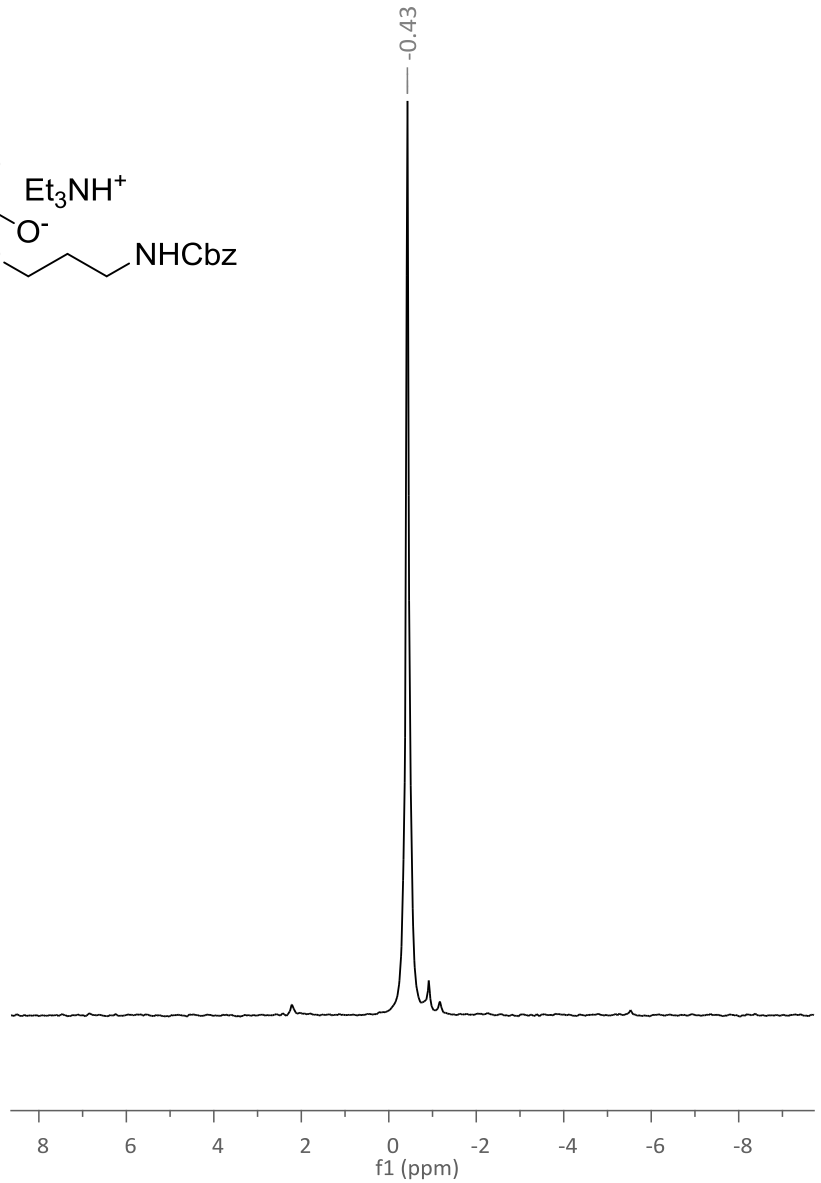

^{13}C NMR (101 MHz, $CDCl_3$) spectrum of compound **9**

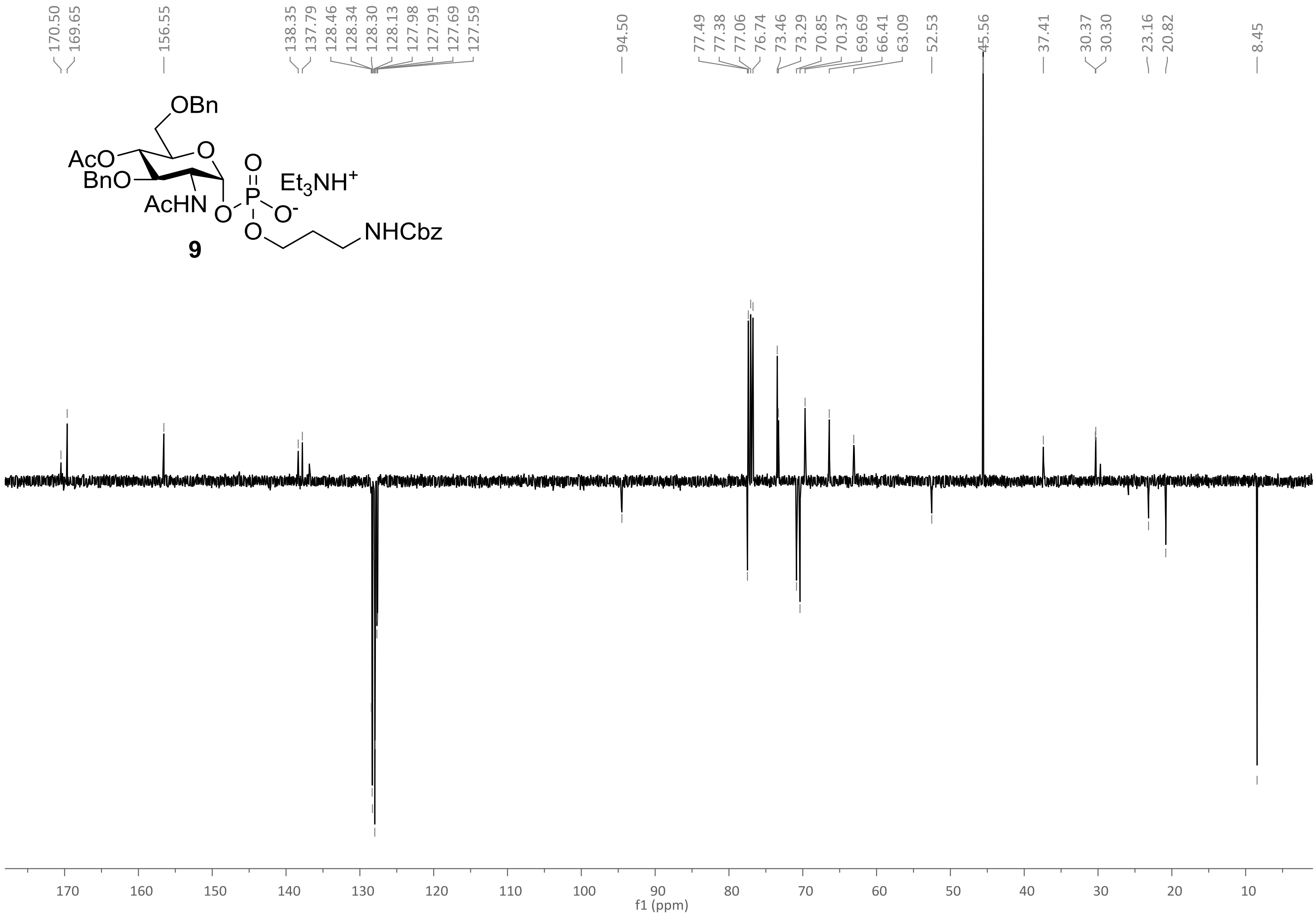

^{1}H NMR (400 MHz, D_2O) spectrum of compound **1**

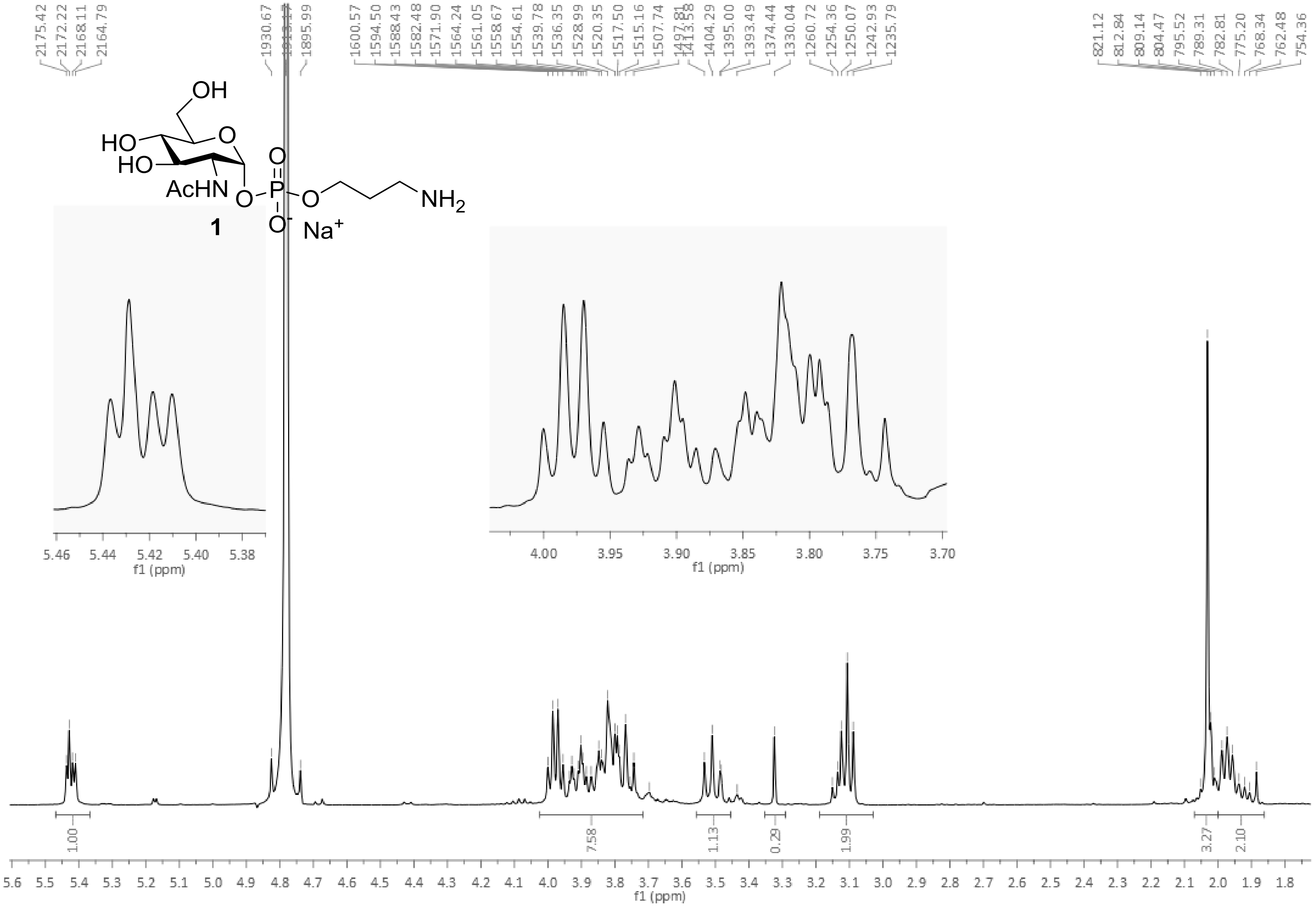

^{31}P NMR (162 MHz, D_2O) spectrum of compound **1**

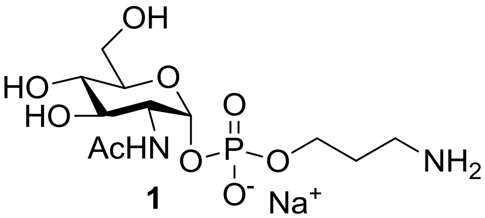

1

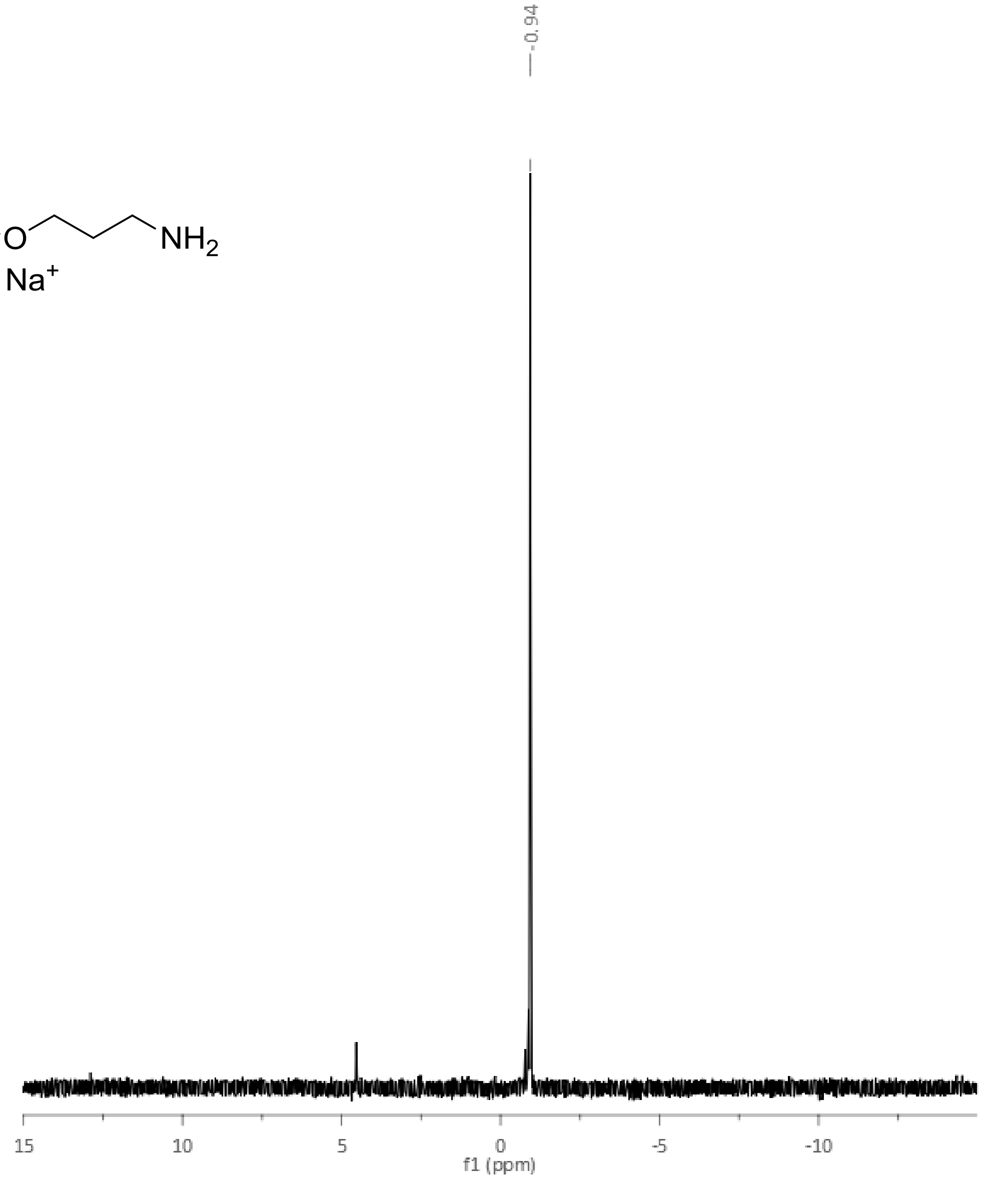

^{13}C NMR (101 MHz, D_2O) spectrum of compound **1**

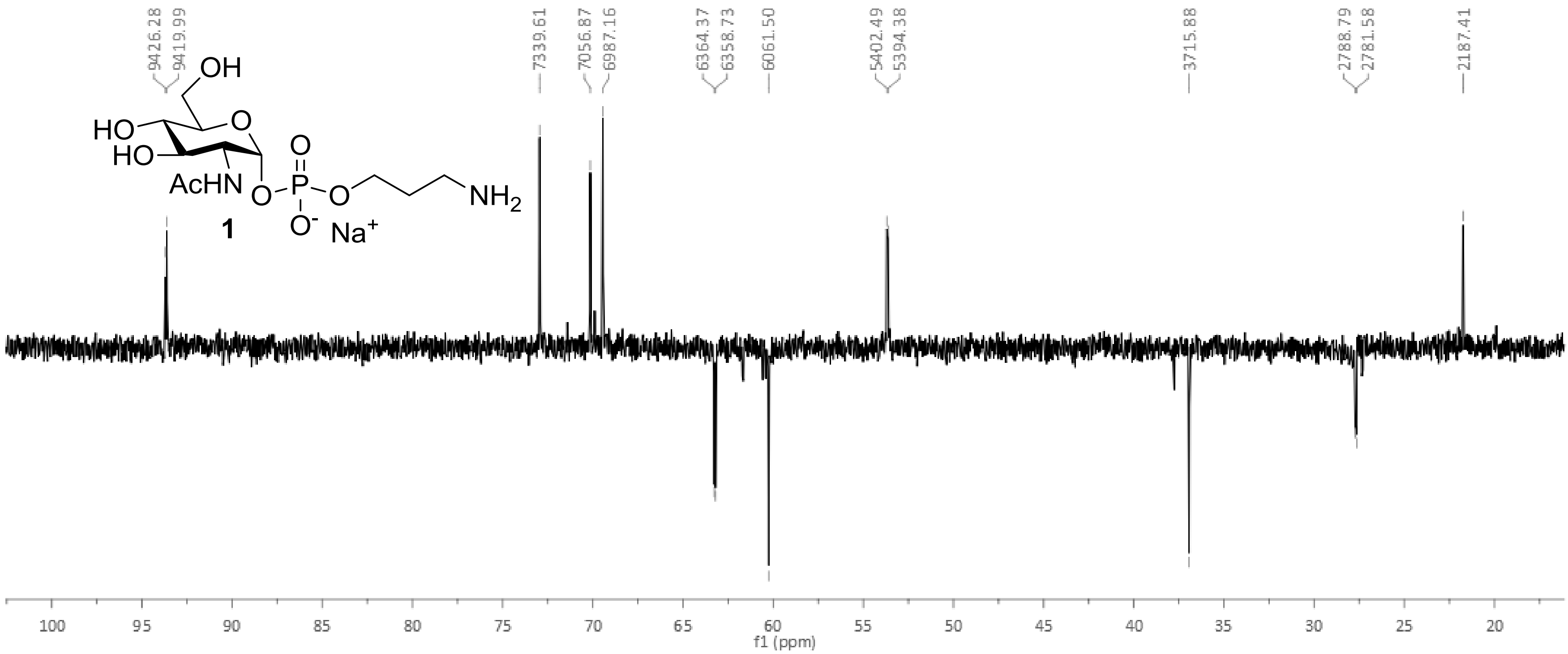

Supplement: File 1 — Experimental procedures for the synthesis of compounds 1, 5, 6, 7, 9, 19, copies of 1H NMR and 13C NMR spectra of compounds 5–6 and 1H NMR, 13C NMR and 31P NMR spectra of compounds 1, 7, 9. [file Beilstein_J_Org_Chem-10-2367-s001.pdf]
